# RNA Regulatory Networks: Key Hubs in the Panorama of Cancer and Emerging Therapeutic Targets

**DOI:** 10.1002/mco2.70586

**Published:** 2026-02-24

**Authors:** Xuan Yin, Zengkan Du, Shuya Jiang, Yan Liao, Changli Wang, Jiaqi Li, Haoling Zhang, Ting‐Ting Wei, Wangzheqi Zhang, Zui Zou

**Affiliations:** ^1^ Faculty of Anesthesiology, Changhai Hospital Naval Medical University Shanghai China; ^2^ Basic Medical University Naval Medical University Shanghai China; ^3^ Department of Gastroenterology, National Clinical Research Center For Digestive Diseases, Changhai Hospital Naval Medical University Shanghai China; ^4^ The Third Department of Hepatic Surgery, Eastern Hepatobiliary Surgery Hospital Naval Medical University Shanghai China; ^5^ School of Anesthesiology Naval Medical University Shanghai China; ^6^ Department of Biomedical Science Universiti Sains Malaysia Penang Malaysia; ^7^ Department of Laboratory Medicine, Shanghai Changzheng Hospital Naval Medical University Shanghai China

**Keywords:** cancer, liquid biopsy, precision medicine, RNA regulatory network, therapeutic target

## Abstract

Cancer is a global health challenge. The initiation and progression of cancer are correlated with dynamic dysregulation of RNA regulatory networks. This review systematically explains how contending RNAs (including mRNA, miRNA, lncRNA, circRNA, etc.) remold gene expression programs across multiple dimensions. They do this primarily through the competing endogenous RNA sponge effect, RNA–protein complex assembly, RNA editing (A‐to‐I editing, m6A modification, etc.), tumorigenesis, heterogeneous evolution, and therapeutic resistance. RNA regulatory networks do not only help one to decode cancer biology but because they are dynamic in nature, they are now also being looked at as good precision targets for diagnosis and treatment. This article integrates recent findings on the emerging functions of RNA networks in tumor metabolic reprogramming, tumor immune microenvironment shaping, and cancer stem cell property maintenance, while highlighting their clinical application prospects as liquid biopsy biomarkers. Our therapies focus on assessing the potential and clinical translation bottlenecks of novel RNA‐targeted interventions, including antisense oligonucleotides, RNA aptamers, and the CRISPR–Cas13 system. A dynamic adjustability made the RNA‐targeted therapies promising intervention nodes in precision medicine even if most of them are still in a preclinical state.

## Graphical abstract

RNA regulatory networks play a crucial role in the initiation and progression of cancer through various modes of RNA interactions. Notably, circulating RNAs have emerged as potential biomarkers, while targeted interventions in RNA regulatory networks facilitate precise therapeutic strategies.

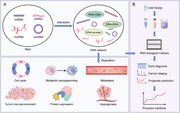

## 1 Introduction

Cancer is a major global health challenge. It has a high morbidity and mortality rate, creating a greater medical need [1, 2, 3]. According to epidemiological data for 2021, 17% of total deaths occurring globally were due to cancer. It causes death rate second higher than heart disease only [4]. In 2022, the number of newly diagnosed cancer cases worldwide approached 20 million, among which approximately 9.7 million were cancer‐related deaths globally [5]. These mortality data show that current treatment approaches have basic limitations in the capacity to completely eradicate tumor cells and prevent relapses and metastases [6]. This highlights the clinical need for new precision therapies that can overcome the hurdles to efficacy in treatment [7]. Presently, the main treatments for cancer are chemotherapy, radiotherapy, and surgery; however, they each have serious limitations [8, 9, 10, 11]. Chemotherapy often fails to eradicate all cancer cells and may cause multidrug resistance, defeating the purpose of the treatment. Moreover, its nontargeted toxic effect destroys the normal tissue thereby resulting in adverse effects like alopecia, nausea, and immunosuppression [8]. Radiotherapy is physically limited by damage to surrounding normal tissues and the inability to kill metastases [9]. In addition, the site of the tumor and state of the patient limit surgical treatment. For cases where the tumor is located on vital organs, or where the stage of the disease is advanced, then surgical feasibility will often be low. Postoperative trauma might hinder the immunity of the body [10]. As a result, there is an urgent need for the development of such precision‐targeted therapeutic strategies in cancer [6]. Clinical treatment hurdles drawn attention toward need for new and more precise therapies [12, 13].

In this context, gaining insights into the biological function of RNA presents renewed hopes to tackle the above‐mentioned therapeutic challenges [14]. As life science research advanced, scientists began to decipher the cellular functions of RNA, leading to a shift in its research scope from merely a “transmitter of genetic information” to a central node in complex‐regulatory networks [15]. Initial research concentrated on studying messenger RNA (mRNA). This RNA acts as a model for the synthesis of ribosomal proteins [16]. Over the past few decades, advances in RNA research have continually increased our understanding of its mechanistic identities, particularly its regulatory functions in tumorigenesis and progression [17]. The discovery of microRNA (miRNA) has marked the beginning of a new era in RNA regulatory research. miRNAs are small RNA molecules that exert their regulatory effect through base complementarity to their target mRNAs, thus allowing a fine‐tuning control of gene expression [18]. Afterward, the discovery of long noncoding RNA (lncRNA) and circular RNA (circRNA) has greatly broadened our understanding of the regulatory ability of RNA [19, 20]. According to a growing body of research, RNA molecules are increasingly proven to regulate oncogenes and tumor suppressor genes [14]. These molecules dynamically regulate the activation of oncogenes and silencing of tumor suppressor genes through RNA–RNA and RNA–protein interactions, activating specific signaling pathways to drive the malignant biological behaviors of tumor cells and enhance their drug resistance [17, 21]. The RNA regulatory network is weaved into essential cellular functions, processes including epigenetic modification, interactions with proteins, and signal transduction pathways (like phosphatidylinositol 3‐kinase [PI3K]/protein kinase B [AKT], Wnt/β‐catenin, etc.) [22]. In addition to the multilevel regulatory mechanisms such as chromosome and RNA (e.g., lncRNA XIST guides the silencing of the entire chromosome by recruiting polycomb repressive complex 2 (PRC2) [23], and circRNA CDR1 acts as an effective sponge of miR‐7 [24]), the functional complexity of RNA is also reflected in its dynamic spatiotemporal regulation. The driving forces behind the various stages of tumor development and progression are critical [23, 24]. These networks play a pivotal role in the evolution of malignant phenotypes, including tumor cell proliferation, metabolic reprogramming, microenvironment remodeling, invasion and metastasis, and angiogenesis [25, 26]. Moreover, RNA regulatory networks are associated with cancer heterogeneity, significantly influencing intratumoral heterogeneity and the biological characteristics of cancer stem cells (CSCs) [27, 28]. Therefore, abnormalities in RNA regulatory networks are closely associated with clinical limiting factors such as chemoresistance, radioresistance, and tumor recurrence, opening up new avenues for cancer treatment [14]. This requires research to move beyond focusing on single RNA types or static interaction modes, and instead shift toward systematic analysis of the dynamic transcriptome–epigenome–proteome network [29]. More importantly, studies have confirmed that a variety of RNA molecules can be applied clinically as diagnostic biomarkers, prognostic indicators, and therapeutic targets [30, 31, 32]. This is gradually advancing the development of precision medicine and providing new strategies for the early detection, individualized treatment, and dynamic monitoring of diseases such as cancer [30, 31, 32, 33]. Future research needs to further verify the clinical utility of these RNAs and develop targeted intervention approaches to reduce the global incidence and mortality of cancer [14]. Currently, a variety of novel RNA‐targeted interventions—including oligonucleotides (ASOs), RNA aptamers, and the CRISPR–Cas13 system—have shown great potential in precision therapy [34, 35, 36]. Nevertheless, they are confronted with significant clinical translation hurdles involving low delivery efficiency, off‐target effects, and immune activation [37, 38, 39]. Interdisciplinary collaboration is urgently needed to facilitate their translation into clinical applications [37, 38, 39].

The article systematically elaborates the important roles of RNA regulatory networks in the mechanisms of cancer occurrence and development, tumor heterogeneity, clinical diagnosis, prognostic evaluation, and therapeutic strategies. This article conducts an in‐depth analysis of the architecture of RNA regulatory networks and studies their intrinsic connections with cancer stages. The article not only reveals the validities of RNA regulatory networks as novel therapeutic targets, but it also predicts the precision medicine of RNA regulatory networks in the future. This article offers fresh ideas for cancer research and clinical therapy.

## 2 | Fundamental Architecture of RNA Regulatory Networks

About 3% of our genome is made up of genes that encode proteins. The rest, was considered “junk DNA” [40]. The Encyclopedia of DNA Elements (ENCODE) project nonetheless showed that as much as 75% of the human genome can be transcribed into RNA, with a large part being made up of ncRNAs. This finding has completely transformed the RNA function research milieu [41]. Through different structural and interactome patterns, RNA molecules have an essential function within cancer regulatory networks and abnormalities in their respective functions mostly activate a tumor and/or help promote its progression.

### 2.1 Diversity and Functions of RNA

mRNAs, as templates for protein synthesis, exhibit structural characteristics and metabolic abnormalities that serve as important molecular markers of cancer. Eukaryotic mRNA's caps and tails are involved in preventing degradation of the mRNA molecule as well as regulating the efficiency of translation initiation [42]. The 5′ and 3′ untranslated regions (UTRs) are key regulatory segments, among which the 3′UTR frequently serves as a bind site for miRNAs to participate in posttranscriptional regulation of target genes [43]. In cancer, the shortening of the mRNA 3′UTR is a common phenomenon. This feature enables mRNAs to evade the negative regulation of miRNAs, leading to the abnormal activation of oncogenes. For example, in triple‐negative breast cancer (TNBC), the oncogenes *JUN* and *NRAS* undergo abnormal polyadenylation, resulting in shortened 3′UTRs. After losing miRNA binding sites, these oncogenes are continuously overexpressed, promoting tumor proliferation and invasion [44]. In addition, the regulation of mRNA translation efficiency is also closely linked to cancer. Some tumor cells enhance the translation initiation efficiency of oncogenic mRNAs (e.g., by upregulating translation factors such as eIF4E), further amplifying oncogenic signals and exacerbating malignant phenotypes [45].

miRNAs are small ncRNAs approximately 20–22 nucleotides in length. Despite comprising less than 0.02% of total cellular RNA, they are among the most extensively studied molecules in cancer regulatory networks [46]. After a series of processing steps, miRNAs bind to members of the argonaute (AGO) protein family to form the RNA‐induced silencing complex (RISC). At this stage, the second to eighth nucleotides at the 5′ end of the miRNA are referred to as the “seed sequence,” which serves as a decisive element for target recognition [47]. The mature RISC mediates mRNA degradation or translation inhibition through base pairing between its seed sequence and complementary sites in the 3′UTR of target mRNAs, thereby achieving precise regulation of gene expression [48]. In cancer regulation, the abnormal expression of miRNAs disrupts the balance of regulatory networks. On one hand, oncogenic miRNAs such as miR‐155 are overexpressed in various cancers thus promoting the propelling and metastasis of tumor cells via inhibition of tumor suppressor genes [49]. On the other hand, the let‐7 family comprises tumor‐suppressive miRNAs that are often downregulated in cancer. This downregulation results in unchecked activity of their target genes and promotes tumorigenesis [50]. The function of miRNAs is cell context dependent; the same miRNA may have opposing functions in different cancers or even within the same cancer type. This complexity poses challenges for miRNA‐targeted cancer therapies.

lncRNAs are RNA molecules longer than 200 nucleotides [51]. Their unique structural features enable specific interactions with DNA, RNA, and proteins, thereby playing an important role in chromatin remodeling, transcriptional regulation, and posttranscriptional processing. Certain lncRNAs work as molecular scaffolds by mediating the spatial assembly of proteins or nucleic acids for certain molecular interactions [52]. In addition, they can recruit chromatin remodeling and modifying complexes to specific loci modifying DNA/RNA methylation status, chromosomal architecture, and epigenetic modifications for gene expression regulation [53]. The regulation of RNA transcription, localization, and stability by lncRNA occurs by binding to mRNAs or acting as ligands for transcription factors to form complexes. In addition to regulating mRNA directly, lncRNAs can also regulate the expression of miRNA target genes by sequestering miRNAs through complementary “seed sequences,” which acts like a sponge. This sponging action relieves miRNA‐mediated target mRNA suppression, particularly in tumors and certain tissues [54]. At this point, lncRNAs increase the expression levels of target mRNAs indirectly, acting as competing endogenous RNAs (ceRNAs). This is one of the main mechanisms lncRNAs use to participate in cancer regulation.

circRNAs are a class of single‐stranded covalently closed RNA molecules that are generated by back‐splicing, which are present from viruses to mammals. The closed‐loop structure of circRNAs makes them highly stable and more resistant to ribonuclease R (RNase R) [55]. circRNAs are reliable on account of their structural features and their presence in bodily fluids (such as blood, saliva, and urine) gives them added advantage as regulators and biomarkers in cancer (both diagnostic and therapeutic) [55, 56]. The core functions of circRNAs include acting as miRNA sponges and regulating protein functions, among which the miRNA sponge effect is the current focus of research [57]. However, studies on circRNA‐mediated cancer regulation still have limitations. On one hand, the expression of most circRNAs exhibits strict tissue and cell specificity, and their functions vary significantly across different cancers, making it difficult to establish a unified regulatory model [58]. On the other hand, the biogenesis mechanism of circRNAs is complex, and the abundance of some circRNAs is much lower than that of their linear RNA precursors. Whether they can effectively compete for miRNAs and exert regulatory effects in vivo remains controversial [59]. Furthermore, whether circRNAs possess direct functions independent of the miRNA sponge effect, and the significance of these functions in cancer, require further research for verification [60].

PIWI‐interacting RNAs (piRNAs) represent a novel class of small ncRNAs, ranging from 24 to 31 nucleotides in length, that typically exert regulatory functions by binding to members of the PIWI (P‐element Induced Wimpy Testis) protein family. Early studies identified PIWI–piRNA complexes as key silencers of transposable elements in germ cells, while subsequent research revealed their additional role in regulating protein‐coding genes [61]. Evidence indicates that piRNAs modulate posttranscriptional networks through piRNA–RNA interactions, similar to miRNA mechanisms, thereby inhibiting the functions of targets such as mRNAs, transcribed pseudogenes, and lncRNAs. Increasingly, aberrant expression of piRNAs and PIWI proteins in various cancers has been documented, highlighting their potential as novel biomarkers and therapeutic targets for cancer diagnosis and treatment [62]. Nevertheless, current cancer research on piRNAs faces numerous challenges. First, the annotation and functional prediction of piRNAs are highly difficult, and the target genes of most piRNAs have not yet been identified. Second, the expression level of piRNAs in somatic cells is low, and whether they can play a core role in cancer regulation needs verification. Third, the specific regulatory network of piRNA–PIWI protein interactions in cancer has not been established, which limits their clinical translational applications [63, 64, 65].

Small RNAs derived from tRNAs (tsRNAs), also known as tRNA‐derived fragments (tRFs), are generated through the specific cleavage of mature or precursor tRNAs by enzymes such as Angiogenin or Dicer. Based on their length and cleavage sites, they are broadly classified into tRNA halves (tiRNAs, ∼30–35 nt) and tRFs (∼18–22 nt) [66]. Once considered simple degradation byproducts, tsRNAs are now recognized as functional molecules involved in translation regulation, epigenetics, and cellular stress responses. In cancer, specific tsRNAs are dysregulated; they promote or inhibit the proliferation and apoptosis of cancer cells by regulating the expression of oncogenes, thereby playing a crucial role in the invasive metastasis and progression of tumors [67]. For example, a 5′‐tRF derived from tRNA–Glu–CTC can increase the activity of the fat mass and obesity‐associated protein (FTO) demethylase, reduce eIF4G1 methylation, inhibit autophagy, and ultimately promote BC proliferation and metastasis [68]. The stability of tsRNAs in body fluids also makes them promising noninvasive biomarkers for cancer diagnosis and prognosis [67].

RNAs derived from small nucleolar RNAs (sdRNAs) are produced from the processing of small nucleolar RNAs (snoRNAs), which primarily guide the modification of ribosomal RNAs (rRNAs). Specific sdRNAs can exert miRNA‐like functions by binding to AGO proteins and inhibiting target mRNAs [69]. Dysregulation of certain sdRNAs is associated with tumorigenesis. Studies have shown that the expression of sdRNA‐93 actively promotes the malignant phenotype of BC by participating in microRNA‐like regulation [70]. A large number of sdRNAs are significantly correlated with characteristics of the tumor immune microenvironment (TIME), such as immunosuppressive markers, CD8+ T cell infiltration, cytolytic T cell activity, and tumor vasculature. A single set of sdRNAs with tumor immune characteristics can also stratify patient survival. These findings indicate that snoRNAs and their derivatives (sdRNAs) represent a prevalent class of noncoding molecular markers for human cancer immunity [71].

The study of tsRNAs and sdRNAs has greatly expanded the repertoire of functional ncRNAs, thus illustrating the complexity of RNA regulatory networks in cancer biology [72]. But more clarification is needed on their roles in cellular signalling pathways and molecular mechanisms. More research should be conducted to thoroughly investigate the tsRNAs and sdRNAs in tumor cells and their targets [73]. The field of tumor biology and tumor genetics can be revolutionized by RNA‐based studies as with our understanding of the function of novel RNAs increasing we may identify novel mechanisms of tumorigenesis.

### 2.2 RNA–DNA Interactions

The remarkable involvement of RNA molecules in cell signaling is becoming extremely clear. They are not just seen as “transmitters of genetic information.” Within regulatory networks that regulate the functioning of the genome, they are playing a great role. The DNA form direct or indirect complex interaction networks that are essential for expression, maintenance of chromatin architecture, and stability of the genome. When these dynamic interactions become disproportional, they may map critically to cancer initiation and progression.

The R‐loop structure exemplifies of RNA–DNA interaction. This structure is naturally generated during transcription, particularly in preference for regions with high GC content, and it plays an essential role in transcriptional elongation and termination, and the establishment of epigenetic marks [74]. Under healthy physiological conditions, the production and removal of R‐loops are kept in a finely tuned dynamic equilibrium. When R‐loops accumulate abnormally, the balance is disturbed and genomic stability is under threat [75]. This genomic instability is closely associated with cancer. An interesting example in BC and ovarian cancer (OC) with mutations in *BRCA1/2* (breast cancer [BC] susceptibility protein 1/2) shows that elevated levels of R‐loops can induce DNA damage, senescence, and cell death and suppress tumor cell proliferation [76]. On the other hand, in prostate cancer (PCa), R‐loops can collaborate with epigenetic regulators to enhance cancer progression. The INO80 chromatin‐remodeling complex is an ATP‐dependent chromatin‐remodeling complex. It is tasked with removing R‐loops from chromatin and preventing transcription‐replication conflict, which protects cancer cells from genotoxic stress and DNA damage. This allows cancer cells to proliferate and survive unlimitedly [77]. The promoter regions of some oncogenes including *MYC* have the potential to form R‐loops on their own. When these R‐loops accumulate abnormally, they can indirectly turn on oncogenes through local epigenetic alterations or DNA breaks, thus promoting cancer [78].

ncRNAs also play a crucial role in RNA–DNA networks. Enhancer RNAs (eRNAs) and promoter‐associated RNAs, which are markers of transcriptional activity, play a key role in long‐range gene regulation. Dysfunction in these traits significantly drives cancer. eRNAs can form molecular scaffolds that recruit and stabilize transcription factors (e.g., YY1) and coactivators (e.g., p300) at enhancers and can significantly amplify transcriptional activation signals [79]. More importantly, eRNAs can recruit chromatin conformation regulators (e.g., cohesin and mediator complexes) to facilitate the formation of spatial loops between enhancers and target promoters, thus physically bringing regulatory elements closer together to efficiently activate the transcription of distal target genes [80]. lncRNAs have been linked to the formation of higher‐order chromatin structures in the nucleus and regulatory 3‐D stability. Dysregulation of these lncRNAs is a critical factor in the reprogramming of cancer genomes. For example, the lncRNA Xist, a fundamental regulator of X‐chromosome inactivation, coats the X chromosome designated for inactivation, recruits silencing factors such as the PRC2, induces repressive histone modifications across the chromosome, and promotes chromatin condensation, ultimately leading to achieving gene silencing [23]. In addition, certain lncRNAs like NEAT1 serve as scaffolds or recruitment platform for the establishment and maintenance of specific topologically associating domains (TADs) or for setting up chromatin interaction networks on a much longer range, which shapes the 3D spatial configuration of the nucleus [81]. Disruption of RNA‐mediated chromatin organization mechanisms occurs in cancers. Oncogenic chromosomal rearrangements, including gene fusions, can disrupt the insulation provided by TAD boundaries, resulting in a phenomenon known as “enhancer hijacking.” When the boundary is compromised, robust enhancers that normally stay in a given TAD activate an oncogene. This process has been demonstrated in T‐cell acute lymphoblastic leukemia and medulloblastoma [82, 83].

The core network regulating gene expression consists of interactions among RNA and DNA. The normal physiological functions of the cell are supported through the maintenance of R‐loop homeostasis, long‐range effect by ncRNAs through gene expression as well as RNA‐mediated higher‐order chromatin structure organization. As cancer begins and evolves, many key links in these complex regulatory networks malfunction: abnormal R‐loop accumulation causes genomic damage; ncRNA dysregulation activates oncogene networks; and 3D genomic structural disorders change oncogenes’ expression patterns. Consequently, a thorough examination of the molecular mechanisms of RNA–DNA interaction will not only help to grasp the biological nature of tumorigenesis but also will provide an important theoretical foundation for the development of novel anticancer strategies.

### 2.3 RNA–RNA Interactions

The molecules of the RNA interact together in RNA–RNA interaction mechanism. This mechanism is quite an essential molecular regulatory mechanism found in various organisms. These different RNA molecules bind to each other, either directly or indirectly to regulate their mutual structures, functions, and biological processes. Interactions that are direct mainly depend on Watson–Crick base pairing for double‐stranded structures. A few important instances of these interactions are the interaction between tRNA and mRNA for the translation of the genetic code and miRNA and mRNA interactions that lead to mRNA degradation. Importantly, numerous classic ncRNAs not only interact directly with other RNA subtypes through base pairing but also facilitate indirect communication via protein intermediaries.

The targeted binding of miRNAs on mRNAs was regarded as a simplistic model of RNA–RNA interactions. miRNAs recognize and bind to complementary sites on target mRNAs, especially in the 3′UTR, and affect mRNA stability and translation [48]. The regulatory mode employed by miRISC on the target mRNAs will depend on the degree of complementarity of miRISC with the complementary sequences, also called the miRNA response elements (MREs). Perfect complementarity typical seen in plants activates endonuclease activity of AGO2 and direct cleavage of target mRNAs [48]. Imperfect complementarity common in animals primarily inhibits translation of mRNA but does not cause its degradation [84]. Numerous studies show that when miRNA expression is not regulated, it is linked to various human diseases including cancer [85]. An example is the let‐7 miRNA family whose members target and regulate prominent oncogenes such as *RAS* and *MYC*. By research, it is shown that the downregulation of let‐7 miRNAs in cells gives rise to the abnormal activation of these oncogenes, thereby driving tumorigenesis and progression [86].

**FIGURE 1:**
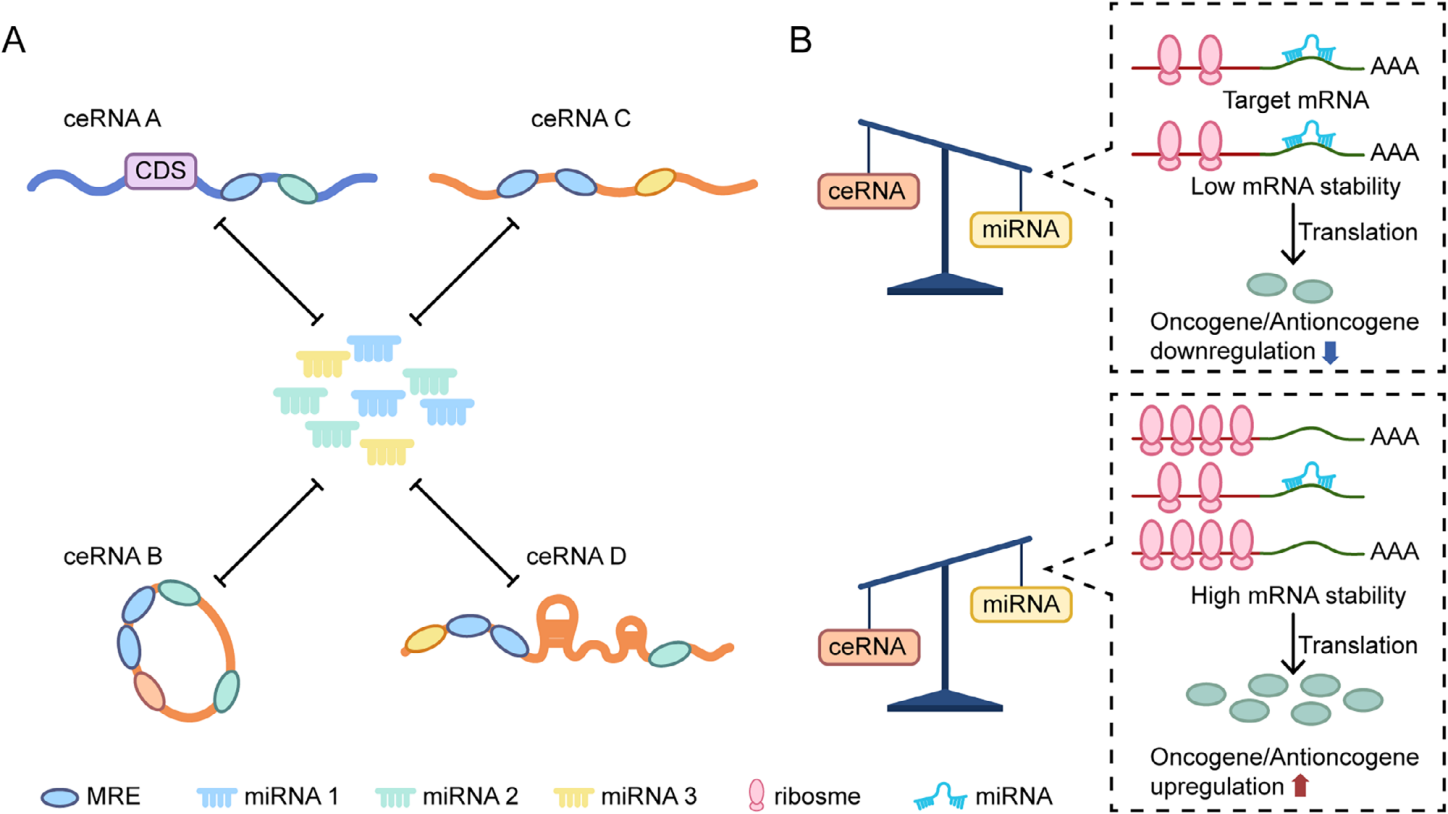
Competitive endogenous RNA (ceRNA) network involving RNA/miRNA/lncRNA/circRNA. (A) miRNAs bind to specific MREs present in both coding (ceRNA A) and noncoding transcripts (ceRNAs B, C, and D) to inhibit gene expression. Transcripts containing MREs for the same miRNA can compete for binding to the shared miRNA pool, thereby reducing the availability of miRNA availability. Transcripts with a greater number of MREs, targeting different miRNAs with varying affinities, may synergistically bind to miRNAs, thereby enhancing their competitive advantage. MREs are depicted as ellipses, whose colors corresponding to their target miRNAs. (B) When the expression level of a ceRNA increases, it functions as a “molecular decoy,” sequestering more miRNAs and resulting in a decrease in the pool of free miRNAs. This diminishes the inhibitory effect of miRNAs on their target mRNAs, leading to an increase in the expression level of the target mRNA. Conversely, a decrease in ceRNA expression results in the release of more miRNAs, which in turn inhibits the expression of target mRNAs. This competitive relationship resembles a “seesaw” effect, where the expressions of ceRNA and target mRNA exhibit an inverse correlation. miRNAs serve as critical regulatory hubs, and their binding preferences are influenced by the relative abundance and affinities of different RNA molecules. This dynamic equilibrium enables cells to finely tune gene expression networks and achieve flexible transcriptional regulation under both physiological and pathological conditions.

The ceRNA network is an essential mechanism in the RNA regulatory networks. The essence of this phenomenon is that different RNA molecules (such as lncRNAs, circRNAs, etc.) share the same MREs, allowing for competitive binding to the miRNAs. This mechanism eases the suppressive control of miRNAs on their target mRNAs, which creates a complex posttranscriptional regulatory layer [87]. In the network, lncRNAs and circRNAs serve as important ceRNA molecules. The expression levels of oncogenes or tumor suppressor genes are dynamically modulated by their ability to sequester miRNAs through a “molecular sponge” effect [88]. The ceRNA network's dynamic equilibrium is manifested in the following form: endogenous upregulation induces ceRNA to serve as “molecular decoys” by sequestering more miRNAs, weakening the inhibition on target mRNAs; conversely, downregulation of ceRNA expression releases more miRNAs, enhancing the effect of silencing on target genes [89]. Gene expression tuning in tumors is commonly involved in this regulatory pattern (Figure 1).

As important parts of the ceRNA network, lncRNAs compete with mRNAs for binding to the miRISC through their miRNA‐binding sites to reverse miRNA‐mediated gene silencing. lncRNA HULC acts as an endogenous sponge sequestering miR‐372 in hepatocellular carcinoma (HCC), thereby relieving miR‐372 inhibition on downstream target genes leading to malignant tumor progression [90]. As a result of their closed circular structure and a high number of miRNA‐binding sites, circRNAs form another type of efficient sponge molecules within the ceRNA network [91]. For instance, CDR1as/circRNA consists of 60 or more conserved miR‐7‐binding sites. As a significant sequestering agent of miR‐7 and the recruiter of AGO protein, it enhances the expression levels of miR‐7 target genes. This plays a very important role in cancer like glioma [24]. Notably, a single circRNA can regulate multiple miRNAs simultaneously. A working circRNA that specifically sequesters miR‐130b/miR‐494 in bladder cancer (BLCA) is circSLC8A1. In cardiac hypertrophy, it primarily binds to miR‐133. This shows the ceRNA function is cell‐ and context‐dependent [92]. According to tissue‐specific miRNA expression profiles, this ceRNA regulatory network may help to reveal tumor heterogeneity and develop tissue‐specific targeting strategies [93].

The ceRNA hypothesis provides a powerful framework for understanding RNA interactions, but it still has some biological limitation such as relative intracellular abundance of miRNAs and ceRNAs, affinity of MREs and subcellular localization [94]. Some studies suggest that the effects of ceRNA regulatory networks may be overestimated in some scenarios whilst false positives are common in experimental validation. This indicates that when analyzing ceRNA networks, it will require comprehensive validation through integration of multiomic data and functional experimentation [95].

Through miRNA‐mediated targeting, lncRNA‐mediated competition, and circRNA‐mediated sponging, RNA–RNA interactions construct a multilevel, multidimensional network for gene expression regulation. The abnormality of these important regulatory systems in major diseases such as cancer, supports their potential as interesting new therapeutic targets. This knowledge helps understand how diseases result as issues arise. It also acts as a basis in developing new therapy strategies. Moreover, the RNA–RNA interaction artificial technologies have moved from the old RNA immunoprecipitation and fluorescence in situ hybridization to the new high‐throughput sequencing ones such as CLASH and LIGR‐seq. They greatly facilitate the systematic dissection of RNA–RNA interaction networks and powerful tools to exploit their roles in biological activities.

### 2.4 RNA–Protein Interactions

**FIGURE 2:**
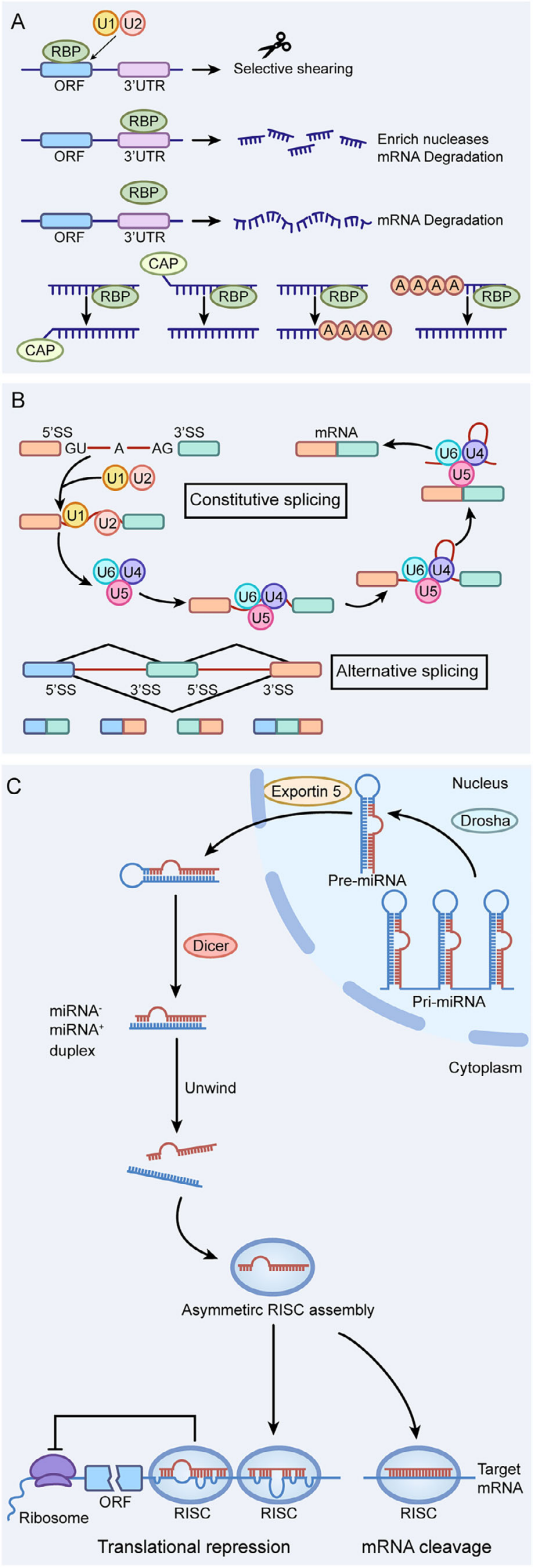
RNA–protein interactions. (A) RNA‐binding proteins (RBPs) bind to target sites within coding regions to promote alternative splicing. Their interaction with the 3′untranslated region (UTR) can either inhibit or induce mRNA degradation or facilitate RNA stabilization; failure of RBPs to bind to target sites in the 3′UTR often results in decreased mRNA stability. Furthermore, RBPs play a crucial regulatory role in significant RNA maturation events, such as polyadenylation and 5′cap addition. They can also initiate RNA degradation processes by recruiting deadenylases to remove the 3′poly‐A tail and decapping enzymes to eliminate the 5′cap structure. (B) RNA splicing primarily encompasses two types: constitutive splicing and alternative splicing. Constitutive splicing represents a fundamental mode of RNA splicing, involving the assembly, rearrangement, and catalytic activity of the spliceosome. The spliceosome consists of five uridine‐rich small nuclear ribonucleoprotein complexes (U1, U2, U4/U6, and U5 snRNPs), which accurately recognize the splice sites of precursor mRNA (pre‐mRNA), completely remove introns, and ligate exons to produce mature mRNA. In this context, splicing changes are limited, resulting in each transcription unit generating only one type of mature mRNA. Alternative splicing occurs when at least one additional 5′splice site (5′ss) or 3′splice site (3′ss) competes with the original splice sites, allowing a single pre‐mRNA to produce multiple mature mRNAs. (C) The maturation of miRNAs and the formation and function of the RISC is a precise multistep process. Initially, miRNA genes are transcribed into primary miRNAs (pri‐miRNAs) with stem–loop structures by RNA polymerase II. Subsequently, pri‐miRNAs are cleaved in the nucleus by the Drosha enzyme and its auxiliary protein, DGCR8, forming precursor miRNAs (pre‐miRNAs). Pre‐miRNAs are then transported to the cytoplasm via exportin‐5, where they are cleaved by the Dicer enzyme to generate miRNA duplexes. The passenger strand of the duplex is degraded, while the guide strand binds to argonaute (AGO) proteins and assembles with additional proteins to form functional RNA‐induced silencing complexes (RISCs). Finally, RISCs regulate gene expression through base pairing between the guide strand and target mRNAs: if the pairing is incompletely complementary, translation is inhibited; if it is completely or nearly completely complementary, mRNA degradation is triggered.

RNA–protein interactions are a molecular regulatory process in living organisms where specific RNA and protein components bind together, thus forming complexes that coordinately regulate gene expression, RNA metabolism, and cell function. Interactions of these types depend primarily on recognition of RNA sequences or secondary structures by protein RNA‐binding domains. Aberrant RNA–protein interactions are closely associated with cancer and thus become crucial targets for disease therapy and RNA‐drug development. Figure 2 illustrates three classical RNA–protein interaction processes.

RNA‐binding protein (RBP) contains multiple RNA‐binding domains and a flexible spatial conformation, plays a central role in regulating RNA metabolism at the posttranscriptional level [96]. RBPs help to maintain transcriptome homeostasis via the regulation of RNA processing and transport. These processes include RNA splicing, polyadenylation, mRNA stability, mRNA localization, and translation [97]. RBPs influence cancer development following key oncogenic events primarily by altering various cancer‐related downstream targets. This modification facilitates a chain reaction that potentiates the phenotypic consequence of the first transformative hit [96]. RBPs function during RNA metabolism in a cell type‐ and context‐dependent manner [98]. The same RBP might have protumorigenic and tumor‐suppressor effect in different cancers, which is challenging for therapy strategies directed against these RBPs [99]. Additionally, current understanding of the interaction networks between RBPs and ncRNAs is still in its preliminary stages, and their dynamic regulatory mechanisms and pathological significance await further exploration [97].

The protein–ribonucleic acid (RNP) complexes formed by interaction of RBP to RNA are the core molecular machinery that regulates gene expression [100, 101]. Research affirms that the abnormal creation of ribosomes—a common kind of RNP complex—is crucial for the onset and progression of most sporadic cancers [102]. The spliceosome is an RNP complex that is made up of small nuclear RNAs and protein factors. It can accurately identify splice sites and catalyze splicing reactions between precursor mRNAs (pre‐mRNAs) [103]. RNA splicing consists mostly of constitutive splicing and alternative splicing [104]. According to alternative splicing, which increases transcript diversity, protein structure and function can be modified diversely. Its dysregulation is closely associated with the occurrence and development of various diseases, especially tumors, and holds significant clinical research value [105].

RISC is a classic study in RNA–protein cooperation. RISC is a complex comprised of small RNAs and members of the AGO protein family that represses target genes. Mature RISC binds to the target mRNAs complementary to the guide RNA. Gene silencing is achieved by cleaving the target mRNAs or recruiting proteins that inhibit the translation and cause degradation of the mRNA. The particular method of action depends on the complementarity of the guide RNA to the target mRNA and on the class of effector molecules recruited by the AGO proteins. In animals, miRNA‐mediated silencing mainly takes place via translation repression and mRNA degradation, with translational repression typically preceding mRNA decay [106]. The degradation of target mRNAs mediated by miRNAs involves processes such as deadenylation, decapping, and exonucleolytic degradation, which are coordinated by the GW182 protein [107]. The mechanisms of translational repression are more complex. They include the dislodging of polyadenylate‐binding proteins, the recruitment of translational inhibitors that target eIF4E or eIF4G and the disassociation of eIF4A [108].

RNA–protein interactions, facilitated by the precise regulation of RNA metabolism by RBPs and gene silencing mediated by RISC complexes, collectively maintain cellular transcriptome homeostasis. These regulatory mechanisms are frequently dysregulated in cancer, a phenomenon characterized by aberrant splicing events and uncontrolled translation. This dysregulation provides a critical theoretical foundation for the development of cancer therapeutic strategies that target RNA–protein interactions.

**FIGURE 3:**
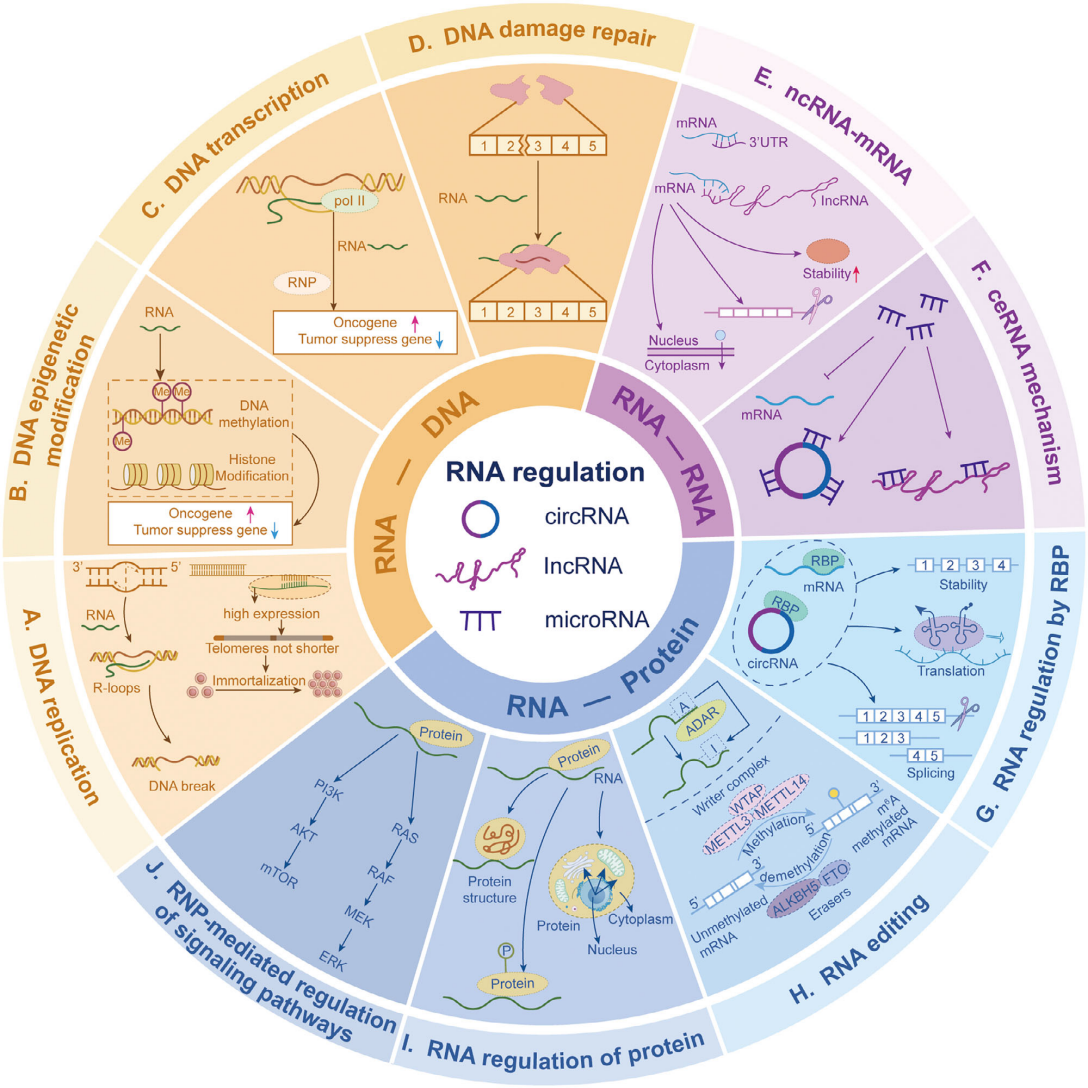
Comprehensive diagram of RNA regulatory network interactions. RNAs interact with DNA, other RNAs, and proteins to form a complex regulatory network. RNA–DNA interactions: (A) DNA replication: RNAs can affect the DNA replication process by forming R‐loops with replicating DNA; increased telomeric RNA extends cell lifespan. (B and C) DNA epigenetic modification/DNA transcription: RNAs or RBPs regulate the expression of oncogenes and tumor suppressor genes by influencing DNA modification and transcription processes. (D) DNA damage repair: RNAs also participate in DNA damage repair. RNA–RNA Interactions (E) ncRNA–mRNA: Through the RNA interference pathway, miRNAs bind to the 3′UTR of target mRNAs, mediating mRNA degradation or translational inhibition. ncRNAs interact with mRNAs via base complementary pairing, affecting mRNA splicing, nuclear export, or stability. (F) ceRNA mechanism: lncRNAs/circRNAs act as “molecular sponges” to sequester miRNAs, relieving the inhibitory effect of miRNAs on target mRNAs. RNA–protein interactions: (G) RNA regulation by RBPs: RBPs bind to specific sequences of RNAs, regulating RNA stability, splicing, or translation. (H) RNA editing: Proteins can modify RNAs to alter their structure and function (e.g., A‐to‐I editing, m6A methylation). (I) RNA regulation of proteins: By binding to proteins, RNAs alter protein conformation, phosphorylation status, or subcellular localization, thereby affecting protein function. (J) RNP‐mediated regulation of signaling pathways: RNAs form complexes with proteins and directly participate in the regulation of cancer‐related signaling pathways.

The unique structure of RNA lays the foundation for its functions, molecular diversity provides the material premise for regulatory networks, and its multidimensional interactions with DNA, other RNAs, and proteins constitute the core link for network formation. These three elements synergistically construct an elaborate regulatory system covering transcriptional, posttranscriptional, and epigenetic levels (Figure 3). Dysregulation of this system's homeostasis is a key driver of cancer initiation and progression, and core regulatory nodes have emerged as potential therapeutic targets due to their specificity and intervenability. However, current research mostly focuses on single molecules or static interactions, lacking systematic analysis of the network's spatiotemporal dynamic evolution and synergistic regulation of multiple components. Future innovative breakthroughs need to break away from the “single‐molecule targeting” mindset and shift toward “global network regulation.” By leveraging cutting‐edge technologies to decipher the dynamic rules of the network, an efficient leap from mechanistic research to clinical translation can be achieved.

## 3 RNA Regulatory Networks and Cancer Onset

Cancer initiation is the result of complex interactions between genetic and epigenetic factors. These factors lead to the abnormal activation of key oncogenes (e.g., *MYC* and *RAS*) and the inactivation of tumor suppressor genes (including *TP53* and *RB1*), ultimately giving rise to cancer‐specific tissue characteristics [109]. RNA regulatory networks play a critical role in cancer initiation through multiple mechanisms, including the activity of noncoding RNAs (e.g., oncogenic miR‐21 and lncRNA HOTAIR), epigenetic modifications, and RNA editing. The subsequent sections will systematically explore these mechanisms from three perspectives: the activation of oncogenes and silencing of tumor suppressor genes, RNA regulation and epigenetic modifications, and the role of RNA editing in cancer initiation.

### 3.1 Oncogene Activation and Tumor Suppressor Gene Silencing

As tumor cells multiply unchecked, they gain the ability to become self‐sufficient in growth signals. This is achieved by activating oncogenes while simultaneously inactivating tumor suppressor genes [110]. Studies from large projects like The Cancer Genome Atlas and the International Cancer Genome Consortium using deep sequencing have shown that mutations that occur in tumors not only happen in protein‐coding sequences but they happen extensively in noncoding regions. Driver mutations in cancerous tumors can occur in various genetic elements, including noncoding. Research established that mutations within the ncRNAs of cancer genome may target oncogenes *TERT* and *MIR‐21*. It also includes tumor suppressor genes *TP53*, *APC*, *BRCA1*, and *RB1* [111]. In addition, evidence is also building that certain ncRNAs may function as oncogenes or tumor suppressors, which are important regulators of human tumor initiation and progression [17].

The importance of miRNAs in oncogenesis has caught a lot of attention. Specific miRNAs are overexpressed in cancer cells. They facilitate cancer progression via tumor cell proliferation, invasion, malignant behavioral promotion, and regulation of development in numerous cancer types. Notably, miR‐126 is significantly overexpressed in BC, colorectal cancer (CRC), and human B‐lymphoblastic leukemia (B‐ALL) [112, 113, 114]. Further studies have demonstrated that the overexpression of miR‐126 downregulates the expression of *TP53* and its associated genes [115]. miR‐155 has been identified as an oncogene in various cancers and is regarded as a therapeutic target for different malignancies [17]. In contrast, miRNAs such as let‐7 function as tumor suppressors, with most being downregulated in various cancer types. Let‐7 miRNAs target and downregulate the expression of multiple oncogenes, including *E2F1*, *ARID3B*, *K‐RAS*, and *C‐MYC*, effectively inhibiting tumor progression [116]. Indeed, some miRNAs can be classified as either oncogenes or tumor suppressors, depending on the cellular context in which they are expressed. For instance, miR‐221 and miR‐222 target the oncogene *KIT* and inhibit the growth of erythroleukemia, thus acting as tumor suppressors in erythroblasts [117]. However, they also target at least four significant tumor suppressor genes—*PTEN*, *P27*, *P57*, and *TIMP3—*and function as oncogenic miRNAs by inhibiting these tumor suppressors in various human solid tumors [118].

A bidirectional regulatory relationship exists between miRNAs and tumor‐related factors. miRNAs can function as either oncogenes or tumor suppressors, thereby contributing to tumorigenesis. Conversely, alterations in tumor suppressors or oncogenes can also lead to dysregulated miRNA expression. These factors can act as transcriptional activators or repressors, directly regulating the transcription of pri‐miRNAs [119]. For instance, the tumor suppressor *p53* can promote the expression of the miR‐34 family, whose members work synergistically with the *p53* network to inhibit abnormal cell proliferation and induce apoptosis by suppressing growth‐promoting genes. During carcinogenesis, *MYC* can activate the expression of oncogenic miRNAs such as the miR‐17∼92 cluster, while simultaneously repressing the transcription of tumor‐suppressive miRNAs in B‐cell lymphoma. Additionally, various cancer‐related transcription factors participate in the aberrant regulation of miRNA transcription [119].

Similar to miRNAs, ncRNAs, including lncRNAs and circRNAs, can function as either oncogenes or tumor suppressors thereby regulating the onset and progression of tumors. These findings establish a critical theoretical foundation for the development of ncRNA‐targeted therapeutic strategies in cancer treatment. Table 1 provides a summary of notable ncRNAs that are of significant interest in cancer research along with their respective functions.

**TABLE 1:** ncRNAs of great concern in cancer research and their functions.

Functional classification	RNA type	Name	Mechanism of action	Cancer types	References
Oncogene	miRNA	miR‐17‐92	Targets and inhibits genes such as *E2F1*, *BIM*, *CREBL2*, *PRRG1*, and *NTN4*, thereby promoting the proliferation and survival of tumor cells	LYM, LC, HA, etc.	[120, 121, 122, 123, 124]
		miR‐125b	Targets tumor suppressor genes (e.g., *BAK1*, *TP53*), and promotes tumor proliferation, metastasis, apoptosis resistance, and therapeutic resistance	BC, BLCA, CC, GBM, LSCC, TC, etc.	[125]
		miR‐21	Targets tumor suppressor genes (e.g., *PTEN*, *TIMP3*), and promotes the proliferation, migration, and invasion of tumor cells	BC, CRC, GC, NA, etc.	[126, 127, 128, 129]
		miR‐221/222	Exerts oncogenic effects by inhibiting the expression of tumor suppressor genes, such as *PTEN*, *P27*, and *P57*. This inhibition promotes tumor progression, regulates the tumor microenvironment, and contributes to chemotherapy resistance.	BLCA, CC, OC, PCa, RCC, etc.	[130]
		miR‐373	Targets genes such as *LATS2*, *PPP6C*, *DKK1*, *TNFAIP1*, and *TP53INP1*, and promotes the proliferation and migration of tumor cells	BC, HCC, etc.	[131]
		miR‐548	Targets genes such as *ABCG2* and *KIF2C*, and is associated with tumor drug resistance	BC, BLCA, PCa	[132, 133, 134]
	lncRNA	ANRIL	Promotes tumor progression mainly by enhancing NF‐kB signaling	GC, HCC, PC, etc.	[135, 136, 137, 138]
		HOTAIR	Interacts with complexes such as PRC2 and LSD1, participates in chromatin remodeling and gene silencing, and promotes tumor occurrence, development, and metastasis	CRC, GC, HCC, PCa, etc.	[139]
		H19	Can act as a host gene for miR‐675 and participate in regulating processes such as cell proliferation, migration, and invasion	BC, CRC, HA, LC, etc.	[140]
		MALAT1	Controls the proliferation, invasion, metastasis, and apoptosis of lung cancer cells through various signaling pathways (e.g., PI3K/Akt and Wnt/β‐catenin)	BC, LC, NPC, etc.	[141]
		PCA3	Upregulates the expression of *PRKD3* through the miR‐1261 sponge effect to promote the progression of PCa cells	PCa	[142]
		PRNCR1	Upregulates the expression of *HEY2* by competitively binding to miR‐448, interacts with androgen receptors, enhances androgen receptor‐mediated gene activation, and promotes tumor cell proliferation	BC, CRC, NSCLC, PCa, etc.	[143, 144, 145]
		PVT1	Affects the expression of *P21* and *YAP*, can sponge various miRNAs such as miR‐31, miR‐503, miR‐551b, etc. This process participates in the regulation of several signaling pathways, thereby affecting cell proliferation, migration, and invasion, while simultaneously inhibiting cell apoptosis.	BC, BLCA, CC, CRC, GC, NSCLC, OC, etc.	[146, 147, 148, 149, 150, 151, 152, 153, 154, 155, 156]
		UCA1	Regulates signaling pathways such as ERK and Wnt/β‐catenin, and promotes the proliferation and survival of tumor cells	BC, CRC, GC, LC, etc.	[157]
	circRNA	circPRKCI	Acts as a miRNA molecular sponge through the ceRNA mechanism, interacts with RBPs as a protein sponge or scaffold, translates into proteins or small peptides, and regulates mRNA alternative splicing, chromatin looping, and gene transcriptional regulation	HCC, GC, LC, TCC, etc.	[158]
		circHIPK3	Sponges and regulates miRNAs of oncogenes or tumor suppressor genes, and participates in the occurrence and development of cancer as a transcriptional regulator	CRC, HCC, OS, etc.	[159]
		circZNF609	Acts as a sponge for multiple miRNAs, and promotes tumor occurrence, development, and radioresistance	BC, CC, GC, HC, PCa, TC, etc.	[160, 161, 162, 163, 164, 165, 166]
Tumor suppressor	miRNA	miR‐15a/16	Inhibits angiogenesis, cell proliferation, cell invasion, and metastasis by inducing apoptosis in cells. Additionally, it increases the sensitivity of tumor cells to chemotherapeutic agents and suppresses tumor growth within in their microenvironment.	BC, CLL, LC, PDAC, etc.	[167]
		miR‐34a	Affects almost the entire process of cancer progression	BC, CRC, LC, etc.	[168]
		miR‐125b	Blocks proliferation and metastasis and enhances therapeutic sensitivity by inhibiting oncogenic target genes (e.g., *HMGA1*, *PIK3CD*)	CRC, GC, EC, HCC, OC, OS, etc.	[125]
		miR‐200	Targets genes such as *ZEB1* and *ZEB2*, inhibits epithelial–mesenchymal transition, thereby suppressing tumor invasion and metastasis	BLCA, GC, PC, PCa, OC, etc.	[169]
	lncRNA	GAS5	Inhibits the proliferation, migration, and invasion of tumor cells while promoting cell apoptosis through multiple mechanisms, such as mimicking GRE, acting as a miRNA sponge, regulating mRNA translation, and affecting protein stability and cyclins	BC, CRC, LC, PCa, etc.	[170]
	circRNA	circMTO1	Acts as a miRNA sponge, inhibits multiple signaling pathways, inhibits cell proliferation, migration, and invasion, and induces cell apoptosis	CRC, GC, HCC, OS, etc.	[171]
		circITGA7	Inhibits the Ras signaling pathway, regulates the miR‐1471/MTDH axis, functions as a molecular sponge for miR‐370‐3p, and suppresses the proliferation and metastasis of tumor cells	CRC, GC, PCa, etc.	[172, 173, 174]
		circZNF609	Improves cancer progression is enhanced and cisplatin sensitivity is inhibited through cascade reactions involving either miR‐1200/CDC25B or circZNF609/IGF2BP2/CD36. Furthermore, it demonstrates that the upregulation of miR‐186‐5p significantly inhibits the growth, migration, and invasion of PCa cells; while also highlighting the upregulation of p53 as a promoter of apoptosis of in CRC cells.	BLCA, CRC, PCa, etc.	[175, 176, 177, 178]

Abbreviations: BC, breast cancer; BLCA, bladder cancer; CC, cervical cancer; ceRNA, competing endogenous RNA; CLL, chronic lymphocytic leukemia; CRC, colorectal cancer; EC, esophageal carcinoma; GBM, glioblastoma multiforme; GC, gastric cancer; HA, hepatoma; HCC, hepatocellular carcinoma; LC, lung cancer; LSCC, laryngeal squamous cell carcinoma; LYM, lymphoma; NA, neuroglioma; NPC, nasopharyngeal carcinoma; NSCLC, non‐small cell lung cancer; OC, ovarian cancer; OS, osteosarcoma; PC, pancreatic cancer; PCa, prostate cancer; PDAC, pancreatic ductal adenocarcinomas; RBP, RNA‐binding protein; RCC, renal cell carcinoma; TC, thyroid cancer; TCC, transitional cell carcinoma.

ncRNAs biological functions are not fixed and invariant but display a strong context‐dependence and functional plasticity. They participate in regulating the dynamic balance of tumor‐related gene expression through diverse regulatory pathways. These molecules not only have the ability to switch between onco‐genic and tumor‐suppressive functions depending on the regulatory microenvironment but also show a crosstalk regulatory pattern in which different ncRNA subtypes target core signaling pathways. In a revolutionary way, this brings to light the evidence of the networked architecture and modular organizational nature of tumor molecular regulation, thereby instilling doubts on the single‐molecule view. Researchers have identified a critical susceptibility factor that can inhibit tumor growth, which has the potential to enhance the effectiveness of a wide range of cancer treatments. Future research needs to shift from targeting isolated molecules to systematic analysis of regulatory networks and functional modules. This will provide a new academic perspective for developing more precise and efficient combination therapy strategies.

### 3.2 RNA Regulation and Epigenetic Modifications

Epigenetics is a field that studies hereditary changes in gene expression that do not involve changes to the DNA sequence. Research studies conducted in the last 10 years have shown that epigenetic regulation is vital in cell growth and differentiation, as well as autoimmune diseases and cancer. DNA methylation, histone modification, and ncRNA‐mediated regulation are the main epigenetic control mechanisms, all of which have become a strong focus of research [179]. This section systematically summarizes recent advances in understanding the role of ncRNAs in tumor epigenetic regulation and elaborates on their underlying mechanisms.

DNA methylation is generally characterized by a methylation modification at the C5 position of cytosine. This occurs mostly at symmetric cytidylyl phosphate guanosine (CpG) dinucleotide regions in mammals [180]. The best‐studied change in cancer epigenetics is CpG island methylation. About 70% of mammalian promoter regions have been found to contain CpG islands, and alteration of their methylation status plays a crucial role in transcriptional regulation. These undergo major changes during malignant transformation. Research has revealed that global hypomethylation is a common feature of malignant cells [181]. According to a recent study, every malignant cell studied to date exhibits global hypomethylation. The DNA methyltransferase (DNMT) family, including DNMT1, DNMT3A, and DNMT3B, mediates DNA methylation by adding methyl groups to cytosine residues without changing the sequence of DNA [182].

lncRNAs exhibit multifaceted roles in regulating DNA methylation. First, through specific binding, lncRNAs can recruit DNMTs or demethylases (TETs) to specific gene loci or hinder their binding. Second, certain lncRNAs, such as lncRNA H19, can indirectly regulate DNMT activity by controlling the levels of DNMT cofactors, such as (SAM and SAH). Third, lncRNAs can regulate the expression levels of DNMTs and TETs at multiple levels, including transcriptional and posttranscriptional stages. These regulatory mechanisms have been validated in developmental biology and disease research, particularly in driving cancer initiation and progression [183]. Notably, lncRNAs can regulate DNA methylation not only in gene promoter regions but also in gene bodies. For example, during transcription in dermatomyositis‐related leiomyomas, lncRNAs recruit DNMT3A to induce methylation of the cathepsin G gene, thereby altering its transcriptional activity [184]. Furthermore, lncRNAs themselves are targets of DNA methylation regulation. For instance, hypomethylation of the promoter region of lncRNA SNHG12 enhances its recognition and binding by the Sp1 transcription factor, leading to aberrant overexpression of this lncRNA in cancer cells and increased resistance to temozolomide [185]. In BC studies, three interaction modes between miRNAs and DNA methylation have been identified [186]: (1) DNMTs methylate miRNA promoter regions, causing epigenetic silencing of tumor‐suppressive miRNAs, which in turn affects key biological processes such as cell proliferation, migration, and apoptosis; (2) miRNAs primarily suppressor miRNAs, target and bind to the 3′UTR of DNMTs via RISC, inhibiting DNMT expression; (3) oncogenic miRNAs exhibit abnormally hypomethylated states in BC cells, promoting their overexpression and tumor progression [186].

Histone modifications are covalent chemical alterations (e.g., methylation, acetylation, phosphorylation, ubiquitination) that occur on specific amino acid residues of histones, the core proteins of chromatin. These modifications dynamically regulate gene transcriptional activity by altering chromatin structure or recruiting epigenetic regulatory factor, such as methylation readers and acetyltransferase complexes [187]. RNA molecules can interact with factors related to histone modifications to participate in regulatory processes. For instance, the upregulation of the histone methyltransferase RIZ1 in HCC cells mediates the enrichment of H3K9me1 at the HOTAIRM1 promoter, thereby regulating cancer cell growth and metastasis through the targeting of miR‐125b [188]. Also, lncRNA GClnc1 binds the histone acetyltransferases WDR5 and KAT2A and acts as a modular scaffold for the WDR5–KAT2A complex to coordinate their localization and the specification of histone modification patterns at target genes such as *SOD2*. This eventually changes the biological behavior of gastric cancer (GC) cells [189].

As research advances, growing evidence indicates that various ncRNAs do not act separately but are instead linked through complex molecular networks in epitopes regulation of tumors. Various classes of ncRNAs, along with DNA methylation and histone modifications, exhibit multilevel, multidimensional regulatory loops. Furthermore, these regulatory loops synergistically pattern the gene expression profiles of tumor cells. In addition, collectively drive tumor initiation, development, and progression. Many potential target and strategic directions for precise diagnosis and treatment of tumors is provided by these findings.

### 3.3 RNA Editing and Cancer Onset

Chemical modifications of RNA have emerged as an essential regulator of gene expression and display the dynamic regulatory profiles typical of DNA and histone modifications. RNA modification at both base and ribose positions is critical to fine‐tuning RNA activity and gene expression programs. The processes of “writing,” “erasing,” and recognition by modification enzymes and binding proteins occur at these positions. RNA editing is a type of posttranscriptional modification that plays a major role in normal physiological processes. Examples are: base editing, methylation, and pseudouridylation. These changes can dynamically control RNA processing, stability, and translational efficiency by altering RNA–protein interaction patterns or RNA secondary structure [190]. RNA editing can change mRNA sequences, leading to differences with genomic templates and causing isoforms of proteins with different functions. Research has shown that aberrant RNA editing can drive the activation of oncogenes or the inactivation of tumor suppressors, a major contributor to cancer initiation and progression [191]. As research continues to advance, the dysregulation of RNA modification pathways is increasingly being implicated in human cancers, making them promising targets for cancer therapy [192].

The A‐to‐I modification is the most widespread type of RNA editing found in metazoans. The adenosine deaminases acting on RNA family (ADAR) facilitates the process [193, 194]. The upregulation of ADAR1 expression or activity has been linked with cancer of GC, BC, liver cancer (HCC), non‐small cell lung cancer (NSCLC), thyroid cancer (TC), and chronic myeloid leukemia (CML). On the other hand, decreased expression of ADAR1 is significantly associated with enhanced development of metastatic melanoma and renal cancer [195]. The ADAR family controls tumor formation using different mechanisms, which includes regulating miRNA maturation, for example, ADAR1 A‐to‐I editing of pri‐let‐7d, which inhibits its maturation and enhances self‐renewal and therapeutic resistance of CML stem cells [196]. Second, ADARs interact with the Dicer protein to promote oncogenic miRNA processing independently of their catalytic activity [197]. Third, they edit the 3′UTR of mRNA to modify miRNA binding sites, thereby influencing the stability of key oncogene mRNAs; for example, ADAR1p110 can competitively inhibit Staufen1‐mediated mRNA degradation [198, 199]. Interestingly, RNA molecules can also reciprocally regulate ADAR function; for example, the immunosuppressive lncRNA LINC00624 stabilizes ADAR1, promoting tumor progression and drug resistance [200].

m6A modification occurs posttranscriptionally in nearly 90% of human mRNAs and ncRNAs, exerting significant biological effects in tumor initiation and progression [201]. m6A is predominantly localized in the 3′UTR and near the stop codon of mRNAs, and is regulated by specific methyltransferases (“writers”), demethylases (“erasers”), and m6A‐binding proteins (“readers”). The core methyltransferase complex comprises methyltransferase‐like (METTL) 3 and METTL14, with METTL3 serving as the catalytic subunit. Other proteins such as Wilms tumor 1‐associated protein, VIR‐like m6A methyltransferase associated, RNA‐binding motif protein (RBM) 15, and zinc finger CCCH‐type containing 13 support this complex, facilitating the cotranscriptional deposition of m6A on nascent pre‐mRNAs. Demethylation is executed by enzymes including FTO and alkB homolog 5 (ALKBH5). Reader proteins that recognize and decipher m6A marks include YTHDF1‐3, YTHDC1‐2, and additional members such as insulin‐like growth factor 2 mRNA‐binding proteins (IGFBPs), musashi2 (MSI2), proline‐rich coiled‐coil containing protein 2A (PRRC2A), and heterogeneous nuclear ribonucleoprotein A2/B1 (HNRNPA2B1) [202]. Writers and erasers maintain a dynamic balance through competitive colocalization and coregulation of expression. Readers recognize m6A via the YTH domain or structural switch mechanisms, dictating RNA fate: YTHDF2 recruits the CCR4–NOT complex to degrade mRNAs, YTHDF1 recruits eIF3 to promote translation, and YTHDC1 regulates splicing and nuclear export [203, 204, 205]. The activity of these three classes of proteins modulates RNA stability, translation, splicing, and localization in a spatiotemporal way that determines the cellular phenotypic output [205, 206]. ncRNAs have also been extensively studied in regulating m6A modification. In relation to miRNA processing, METTL14, a methyltransferase responsible for m6A, promotes the m6A modification and maturation of pri‐miR‐126 through its interaction with DGCR8, while miR‐126 can antagonize METTL14's inhibitory effect on tumor metastasis [207]. The impact of m6A modification on lncRNAs may involve multiple regulatory mechanisms. On one hand, m6A modification can regulate lncRNA function by providing binding sites for m6A reader proteins or by adjusting local RNA structures to facilitate the entry of RBPs. On the other hand, m6A modification may also influence the relationship between lncRNAs and specific DNA sites by affecting RNA–DNA triple helix structures. In glioblastoma (GBM) stem cells, the m6A demethylase ALKBH5 interacts with lncRNA FOXM1‐AS to promote cancer cell proliferation and tumorigenicity [208]. Additionally, m6A modification of circRNAs can recruit eIF4G2 and YTHDF3, facilitating the translation process of circRNAs [209]. The functional heterogeneity of m6A modification mainly stems from three factors: site topology and structural context, cancer type, and the competitive interaction of reader proteins [210, 211]. Current controversies focus on the “bidirectional paradox” of writers such as METTL3—their protumor or tumor‐suppressive activity is not an intrinsic property but conferred by the cell type‐specificity of the target transcriptome profile [212]. Research limitations are reflected in insufficient technical resolution and sensitivity, fragmented mechanistic studies (i.e., neglecting the synergy of multiprotein complexes and cross‐talk between different modifications), and lack of evidence for clinical translation [213, 214, 215]. In the future, it is necessary to integrate single‐cell multiomics and spatiotemporal transcriptomics to construct causal RNA interaction network models, thereby deepening the understanding of m6A [213].

5‐Methylcytosine (m5C) modification refers to a methylation modification at the C5 position of the cytosine ring in RNA [216]. m5C is enriched in 3′UTRs, GC‐rich sequences, and regions near start codons, where it regulates mRNA stability, nuclear export, translation, and DNA damage repair [217]. Similar to m6A, dysregulation of m5C modification leads to altered RNA stability, disrupted splicing patterns, and changed epigenetic profiles [218]. It also impairs normal RNA folding, modifies RNA interactions with other molecules, affects the subcellular localization of specific RNAs, regulates the expression of immune‐related genes and immune checkpoints, and may influence the tumor microenvironment (TME) and immune escape mechanisms [218]. In epidermal growth factor receptor‐NSCLC, aberrant RNA m5C modification mediates intrinsic resistance to gefitinib through the NSUN2–YBX1–QSOX1 axis [219]. The tumor‐promoting role of m5C is not limited to mRNA; many m5C‐modified ncRNAs also play critical roles in tumorigenesis. For example, NSUN2 enhances the stability of the lncRNA NKILA in a YBX1‐m5C‐dependent manner. As a sponge for miR‐582‐3p, NKILA promotes the expression of YAP1, thereby driving the development of cholangiocarcinoma [220].

The three RNA modifications mentioned above have been extensively studied in the field of cancer. The latest advances in their effects on tumors are summarized in Table 2.

**TABLE 2:** Recent advances in the impact of RNA editing on tumors.

RNA editing	Editing enzyme	Cancer	Target factor	Impact	References
A‐to‐I	ADAR1	BC	ARPIN↑	Inhibits the mobility of BC cells	[221]
	ADAR1	BC	MDM2↑	Oncogenic	[222]
	ADAR1	BC	METTL3↑	Promotes the proliferation, migration, and invasion of cancer cells	[223]
	ADAR1	BLCA	miR‐154‐p13‐5p↑	Targets LIX1L to inhibit cancer cell proliferation and migration, and induce cell apoptosis	[224]
	ADAR1	CRC	AZIN1↑	Upregulates IL‐8 to promote tumor angiogenesis	[225]
	ADAR1	CRC	BLCAP↓	Reduced levels of BLCAP protein lead to the loss of its inhibitory effect, thereby promoting the transition of the cell cycle from the G1 phase to the S phase. This transition results in increased cell proliferation and decreased apoptosis.	[226]
	ADAR1	GC	miR‐302a↑	Inhibits interferon signaling in gastric cancer cells	[227]
	ADAR1	GC	SCD1↑	Drives chemoresistance and self‐renewal of tumor cells	[228]
	ADAR1	ESCC	USP38↑	Promotes cell proliferation	[229]
	ADAR1	HA	miR‐3144‐3p↑	Enhances the expression of oncogenic RNA‐binding protein 2	[230]
	ADAR1	ICC	BRCA2↑	Promotes tumor progression and DDP resistance in ICC	[231]
	ADAR1	LUAD	miR‐1251‐5p↑	Regulates the TCF7‐mediated Wnt signaling pathway to inhibit tumor growth and metastasis	[232]
	ADAR1	NSCLC	CYP1A1↑	Drives lung carcinogenesis	[233]
	ADAR1	NSCLC	miR‐411‐5p↑	Promotes the response of NSCLC‐resistant cells to tyrosine kinase inhibitors	[234]
	ADAR1	PCa	POLA2↑	Exerts oncogenic effects by impeding immune infiltration, enhancing glycolysis, and upregulating BTBD7 expression	[235]
	ADAR1	PDAC	circNEIL3↓	Regulates circNEIL3 through a negative feedback loop, attenuating the EMT effect	[236]
	ADAR1	TC	CDK13↑	Oncogenic	[237]
	ADAR1 and ADAR2	OS	EMP2↑	Eliminates the inhibitory effect of miRNAs on target factors	[238]
m6A	METTL3	BLCA	CXCL5↑, CCL5↓	Promotes the formation of an immunosuppressive microenvironment	[239]
	METTL3	CRC	CTNNB1↑	Regulates antitumor immune response	[240]
	METTL3	CRC	LINC02418↑	Promotes proliferation and metastasis	[241]
	METTL3	CRC	MMP9↑	Promotes proliferation and migration	[242]
	METTL3	EC	CEP170↑	Promotes cell proliferation and mitosis	[243]
	METTL3	GC	FNTA↑	Drives gastric cancer progression	[244]
	METTL3	HA	BFSP1↑	Induces aerobic glycolysis, promoting the growth and metastasis of liver cancer	[245]
	METTL3	HCC	BMI1 and RNF2↑	Promotes cell proliferation, colony formation, and metastasis	[246]
	METTL3	LSCC	WISP1↑	Accelerates the viability, invasion, migration, and EMT of LSCC cells	[247]
	METTL3	OSCC	miR‐146a‐5p↑	SMAD4, the target of miR‐146a‐5p, is a central transducer of TGF‐β signaling, exacerbating oncogenic traits in OSCC.	[248]
	METTL3	PC	circCEACAM5↑	Inhibits apoptosis while promoting cell proliferation, invasion, and migration	[249]
	METTL3	TC	LINC00969↑	METTL3 levels are downregulated in TC, and the silencing of METTL3 partially reverses the inhibitory effect of LINC00969 overexpression on the malignancy of PTC cells.	[250]
	METTL3	TC	hsa_circ_0136959↑	Overexpression of hsa_circ_0136959 increases the levels of ferroptosis‐related markers in cells.	[251]
	METTL3/YTDHF1	AML	CSRP1↑	Promotes cell proliferation and glycolysis	[252]
	METTL3/YTDHF1	EC	FOXD2‐AS1↑	Promotes cell proliferation and inhibits apoptosis	[253]
	METTL3/YTDHF1	NA	MTCH2↑	Inhibits ferroptosis and promotes tumor cell survival	[254]
	METTL3/YTHDF1	PCa	circRPS6KC1↑	Inhibits cellular senescence	[255]
	METTL3/IGF2BP1	TNBC	PRMT7↑	PRMT7 regulates the Wnt/β‐catenin pathway, promoting cell proliferation, invasion, migration, and glycolysis.	[256]
	METTL3/IGF2BP2	HB	YWHAE↑	Increases the levels of lipid ROS and peroxides in HB cells, enhancing the susceptibility of HB cells to ferroptosis	[257]
	METTL3/IGF2BP2	PCa	circQKI↑	Reduces tumor sensitivity to docetaxel	[258]
	METTL3/IGF2BP3	CC	NFAT5↑	NFAT5 promotes cell growth by transcriptionally regulating PRDX1 expression.	[259]
	METTL13	PCa	CD44↑	Promotes the proliferation of cancer stem cells and enhances the resistance of PCa to docetaxel	[260]
	METTL14	BC	E2F1↑	Promotes CDK4/6 inhibitor resistance	[261]
	METTL14	EC	FSP1↑	Regulates ferroptosis and attenuates DDP resistance	[262]
	METTL14	GC	ATF5↓	Attenuates cancer stemness by inhibiting the ATF5/WDR74/β‐catenin axis in gastric cancer	[263]
	METTL16	AML	TCF‐1↓	Deficiency of TCF‐1 promotes T cell exhaustion and inhibits T cell self‐renewal capacity.	[264]
	METTL16	GC	UBXN1↑	Oncogenic	[265]
	METTL16/IGF2BP2	HCC	LAMA4↑	LAMA4 binds to COL4A1 and promotes DDP resistance and cancer progression through COL4A1.	[266]
	RBM15	BC	KPNA2↑	Drives cancer cell progression and immune escape	[267]
	RBM15	CRC	HUNK↑	SLERT is significantly upregulated in CRC tissues and interacts with RBM15, thereby impairing RBM15's capacity to stabilize HUNK mRNA, which in turn promotes liver metastasis.	[268]
	RBM15	CRC	lncRNA FGD5‐AS1↑	FGD5‐AS1 promotes HOXC10 expression by recruiting YBX1 to the HOXC10 promoter, enhancing CRC cell proliferation.	[269]
	RBM15	GC	ACLY↑	Drives lipogenesis and exacerbates malignant characteristics in GC	[270]
	RBM15	LUAD	SPOCK1↑	Promotes EMT‐mediated osimertinib resistance	[271]
	RBM15	LUAD	XPR1↑	Promotes malignant progression of cancer	[272]
	RBM15	NSCLC	CBR3‐AS1↑	Promotes radioresistance	[273]
	WTAP	CRC	SOX1↓	Promotes the proliferation and tumor growth of CRC cells	[274]
	WTAP	GC	MAP2K6↑	Promotes the proliferation, migration, and invasion of GC cells	[275]
	KIAA1429	GC	LINC0968↑	Promotes cell growth and metastasis	[276]
	PRMT5	CRC	ALKBH5↑	Promotes immune evasion	[277]
	ZCCHC4	EC	ROS↓	Promotes cancer cell proliferation, inhibits apoptosis, and increases cancer cell resistance to DDP	[278]
	FTO	HSCC	SERPINE1↑	Promotes EMT in hypopharyngeal cancer cells	[279]
	FTO	LUAD	QPCT↑	QPCT promotes tumorigenesis in LUAD by increasing macrophage recruitment and the proportion of M2 macrophages	[280]
	FTO	NSCLC	KCNAB2↓	Promotes tumor characteristics and M2 macrophage polarization	[281]
	FTO	OC	NLRP3↓	Increases cancer cell sensitivity to DDP and inhibits tumorigenesis	[282]
	FTO	pNET	APOE↑	Enhances lipid metabolism and proliferative capacity	[283]
	FTO	TNBC	CEBPB↑	Promotes cell proliferation, colony formation, and metastasis	[284]
	ALKBH1	OS	TRAF1↓	Promotes osteosarcoma proliferation and metastasis	[285]
	ALKBH5	GC	TUG1↑	Promotes proliferation, invasion, migration, and angiogenesis	[286]
	ALKBH5	GC	WRAP53↓	Reduced levels of WRAP53 levels disrupt the interaction between USP6 and RALBP1 proteins, leading to the degradation of RALBP1. This process thereby inhibits the PI3K/Akt/mTOR signaling cascade, ultimately attenuating the progression of GC.	[287]
	ALKBH5	NSCLC	XBP1↑	Promotes NSCLC cell proliferation, migration, and invasion by upregulating XBP1 expression and activating IL‐6–JAK–STAT3 signaling	[288]
	ALKBH5	OC	SRSF10↑	Promotes cell proliferation and migration	[289]
	YTHDF	GC	IRF1↓	Leads to downregulation of IFN‐γ signaling, weakening cancer cell immunity	[290]
	YTHDF1	BLCA	RIG‐I↓	Attenuates immune response and promotes cancer progression	[291]
	YTHDF1	CRC	DDX21↑	Regulates lipid metabolism to promote CRC progression	[292]
	YTHDF1	PC	SF3B2↑	Promotes cell proliferation and invasion	[293]
	YTHDF2	GC	AC026691.1↓	Promotes cancer cell proliferation, migration, EMT, and M2 macrophage polarization	[294]
	YTHDF2	GC	ONECUT2↓	Inhibits cell migration, stemness, and oxaliplatin resistance	[295]
	YTHDF3	BC	Notch2↑	Promotes cell migration, invasion, and metastasis	[296]
	YTHDF3	TNBC	CENPI↑	Enhances proliferative and migratory capacities	[297]
	YTHDF3	GC	EZRIN↑	Contributes to GC cell motility and response to paclitaxel	[298]
	YTHDC1	LSCC	lncRNA MSC‐AS1↑	MSC‐AS1 blocks the expression of CTCF, thereby inhibiting the antitumor effect of CTCF	[299]
	IGF2BP2	BC	FGFR2↑	Maintains cell stemness, invasive traits, and angiogenesis	[300]
	IGF2BP2	BLCA	HMGA1↑	Enhances stemness phenotype to promote chemoresistance	[301]
	IGF2BP2	NSCLC	SLC7A11↑	Promotes cell proliferation and inhibits ferroptosis	[302]
	IGF2BP2	PC	SLC1A5↑	Promotes glutamine uptake by PC cells and accelerates cancer progression	[303]
	IGF2BP3	BLCA	CDK6↑	Promotes tumor cell proliferation and resistance to DDP chemotherapy	[304]
	IGF2BP3	BLCA	HSP90AB1↑	Promotes the development of bladder cancer	[305]
	IGF2BP3	HCC	MCM10↑	Enhances cell proliferative and migratory capacities	[306]
	LRPPRC	BC	CXCL11↓	Facilitates BC cell viability, migration, and invasion; overexpression promotes malignant phenotypes	[307]
	HNRNPC	NSCLC	TFAP2A↑	Promotes the malignant progression and EMT of NSCLC	[308]
	PRRC2A	CRC	CSNK1E↑	Promotes cancer progression	[309]
	RBFOX2	OSCC		Enrichment analysis indicates that RBFOX2 is involved in the receptor tyrosine kinase signaling pathway and the Hippo signaling pathway, playing a crucial role in oral carcinogenesis and development. Clinically, elevated expression of RBFOX2 correlates with advanced tumor stages and poor patient prognosis, thereby demonstrating its prognostic significance	[310]
m5C	NSUN1 (NOP2)	HCC	LMNB2↑	Drives the growth and metastasis of CRC tumors	[311]
	NSUN1 (NOP2)	LC	EZH2↑	Mediates EMT	[312]
	NSUN2	ATC	SRSF6↑	Drives alternative splicing reprogramming and promotes multidrug resistance in anaplastic thyroid cancer	[313]
	NSUN2	CC	LRRC8A↑	Promotes proliferation and metastasis	[314]
	NSUN2	CRC	SKIL↑	Promotes cancer cell growth	[315]
	NSUN2	ENCA	SLC7A11↑	Confers ferritin deposition resistance	[316]
	NSUN2	ESCC	LIN28B↑	Enhances the initiation and progression of ESCC	[317]
	NSUN2	GC	NTN1↑	Promotes perineural invasion in gastric cancer	[318]
	NSUN2	HCC	lncRNA H19↑	Causes poor differentiation	[319]
	NSUN2	LC	circFAM190B↑	Inhibits cell autophagy to promote lung cancer progression	[320]
	NSUN2	NSCLC	NRF2↑	Drives ferroptosis resistance	[321]
	NSUN2	NSCLC	QSOX1↑	Induces resistance to gefitinib	[219]
	NSUN2	PCa	TRIM28↑	Promotes the development of prostate cancer	[322]
	NSUN2/YBX1	BC	HGH1↑	Promotes breast cancer progression	[323]
	NSUN2/ALYREF	UBC	RABL6/TK1↑	Promotes malignant development	[324]
	NSUN3	NSCLC	PD‐L1↓	Promotes immune escape	[325]
	NSUN4	NSCLC	CDC20↑	Promotes cancer development	[326]
	NSUN5	CCA	GLS↑	Promotes cancer progression	[327]
	NSUN5	PCa	ACC1↑	Promotes fatty acid synthesis and lipid accumulation	[328]
	NSUN6	CC	NDRG1↑	Induces radioresistance	[329]
	ALYREF	EC	TBL1XR1 and KMT2E↑	Induces oxaliplatin resistance	[330]
	ALYREF	HCC	EGFR↑	Oncogenic	[331]
	ALYREF	ICC	IDH1↑	Promotes cancer progression	[332]
	YBX1	ICC	STAT1↑	Inhibits antitumor T cell response to promote tumor progression	[333]
	YBX1	LSCC	PFKFB4↑	Promotes cancer cell development	[334]

Abbreviations: AML, acute myeloid leukemia; ATC, anaplastic thyroid cancer; BC, breast cancer; BLCA, bladder cancer; CC, cervical cancer; CCA, cholangiocarcinoma; CRC, colorectal cancer; DDP, cisplatin; EC, esophageal carcinoma; EMT, epithelial–mesenchymal transition; ENCA, endometrial cancer; ESCC, esophageal squamous cell carcinoma; GC, gastric cancer; HA, hepatoma; HB, hepatoblastoma; HCC, hepatocellular carcinoma; HSCC, hypopharyngeal squamous cell carcinoma; ICC, intrahepatic cholangiocarcinoma; IL, interleukin; LUAD, lung adenocarcinoma; NA, neuroglioma; NSCLC, non‐small cell lung cancer; OC, ovarian cancer; OS, osteosarcoma; OSCC, oral squamous cell carcinoma; PC, pancreatic cancer; PCa, prostate cancer; PDAC, pancreatic ductal adenocarcinoma; pNET, pancreatic neuroendocrine tumor; ROS, reactive oxygen species; TC, thyroid cancer; TNBC, triple‐negative breast cancer; UBC, urothelial bladder cancer.

Pseudouridine (Ψ) modification is the result of a 180° rotation of the uracil (U) pyrimidine ring around the C3–C6 axis. The base of Ψ is connected to ribose through a C─C bond unlike U whose linkage is through an N─C bond. The orientation of the Ψ base is more stable than that of U, and its N1 position can act as a proton donor and form additional hydrogen bonds that stabilizes RNA [335]. Modification of Ψ impacts cancer development by governing translation or RNA stability [217]. Regarding ribosomes, Ψ modification affects both ribosomal proteins and rRNA. Ψ modification in rRNA increases ribosomal translation efficiency, promotes protein synthesis, and ultimately enhances cell proliferation and invasion [336]. However, miswiring Ψ modifications in particular portions of rRNA may result in miscalculation and tumorigenesis [217]. mRNA molecules that code for Ψ‐modified ribosomal proteins are more stable, which results in a greater abundance of ribosomal proteins and this in turn enhances the proliferation of tumor cells [337]. Moreover, tRNA Ψ modifications impact tumorigenesis by regulating cellular translation. For example, the overexpression of *PUS7* leads to increased Ψ modification on tRNA that inhibits the translation of *TYK2* mRNA and promotes GBM tumorigenesis [338]. Importantly, the Ψ modification plays essential roles in mitochondrial protein synthesis as well as oxidative phosphorylation; enhancement of mitochondrial function may help meet the high energy demands of fast‐growing tumor cells [339].

N^4^‐acetylcytidine (ac^4^C) modification is currently the only known RNA acetylation event occurring on rRNA, tRNA, and mRNA in eukaryotic RNA [340]. N‐acetyltransferase 10 (NAT10) is the only known enzyme that mediates the mRNA ac^4^C modification, which is essential for mRNA stability and translation [341]. According to the research, NAT10 catalyzes the ac^4^C modification of mRNA that activates the overtranslation of oncogenes to enhance cancer cell proliferation, migration, and chemoresistance. The oncogenes that were modified by ac^4^C have the ability to either activate or inhibit key signaling pathways associated with cancer progression. They were also able to regulate the expression of a series of downstream genes [342]. NAT10 further regulates the lipid metabolism in cancer cells through the ac^4^C modification of genes related to fatty acid metabolism [343].

N1‐methyladenosine (m1A) modification is understudied when compared with other RNA modifications and was only recently discovered. New findings suggest that when m1A is not regulated properly, it could change the stability of some types of RNA, the translation of some types of mRNA, RNA processing and maturation, RNA protein interaction, and the deposition and removal of m1A—ultimately contributing to cancer initiation and progression [344]. Building on the successful cases of m6A, m1A modification may provide new strategies and targets for tumor immunotherapy. Researchers need to address challenges such as the scarcity of novel modifications and the promiscuous substrate specificity of many mRNA modifiers. Future research must clarify the roles of regulator m1A in gene and protein regulation, which are likely to be shared with m6A. It must also validate the importance of m1A modification [345].

RNA editing is not an isolated phenomenon; rather, it works in concert with epigenetic regulation, posttranscriptional networks, and the TME to contribute to tumor heterogeneity and therapeutic resistance. ADAR1‐mediated A‐to‐I editing, for example, regulates tumor stemness through the maturation of various miRNAs, including let‐7, and alters the interferon pathway via the editing of immune‐related transcripts, ultimately mediating immune escape [200, 346]. m6A changes enhance the stability of mRNAs from important immune checkpoint molecules promoting immune evasion of tumor cells [347, 348]. There is also m6A and A‐to‐I editing cross‐regulation. As an illustration, by editing METTL3 mRNA, the ADAR1 changes the binding site to miR532‐5p; hence, increasing METTL3. This further targets ARHGAP5, which is recognized by YTHDF1 and promotes further proliferation, migration and invasion of BC cells [223]. Additionally, m1A and m6A modifications share coregulatory proteins, such as members of the ALKBH family and FTO family [349]. Going forward studies will benefit from a systems biology view to explore the interactions (synergistic and antagonistic) among different RNA modifications across spatiotemporal scales and their global roles in the adaptive evolution of tumors.

## 4 Dynamic Changes of RNA Regulatory Networks in Cancer Progression

RNA regulatory networks show a remarkably dynamic spatiotemporal specificity in the context of cancer progression. Their functions extend beyond the regulation of individual genes or pathways; they also drive malignant biological behaviors. Their activities include those that increase tumor cell proliferation, metabolic reprogramming, TME remodeling, invasion and metastasis, and angiogenesis. This section will explore the core regulatory mechanisms of RNA molecules involved in these critical biological events and their potential value in translational clinical applications.

### 4.1 Tumor Cell Proliferation

The cell cycle refers to the regulated sequence of events that leads to cell growth, division, and DNA damage repair [350]. The initiation and progression of the cell cycle depend on the functions of a number of key regulatory factors such as cyclin‐dependent kinases (CDKs), cyclins (CCNs), cyclin‐dependent kinase inhibitor and E2F transcription factors [351, 352]. Cancer‐related mutations frequently affect processes that lead to cell cycle exit, enabling tumor cells to gain a capacity for unlimited division [353].

ncRNAs form intricate regulatory networks by targeting essential cell cycle regulators and associated signaling pathways. It will play the most critical roles in the initiation along with the progression of a cancer. Regarding the influence of ncRNAs on cell cycle regulators, miRNAs can negatively regulate cell cycle progression by inhibiting the expression of cyclins and interfering with transitions between cell cycle phases [354]. Notably, miR‐223, miR‐186‐3p, and miR‐186‐5p have been shown to inhibit the proliferation of lung adenocarcinoma (LUAD) and NSCLC cells by targeting CDK2, respectively [355, 356]. Additionally, miR‐613, miR‐545, and miR‐486‐3p exert inhibitory effects on NSCLC by regulating CDK4 [356, 357]. A primary target of lncRNAs in tumor progression is the CDK4/6–CCND1 complex [358]. As core regulatory molecules of the G1/S checkpoint, aberrant activation of CDK4/6 is closely linked to tumorigenesis and progression [359, 360]. The significant downregulation of lncRNA TUG1 in BC tissues results in the upregulation of CCND1 and CDK4, thereby promoting the cell cycle progression of BC cells [361]. Furthermore, lncRNAs and circRNAs can indirectly influence cyclin‐mediated cell cycle regulation through interactions with miRNAs. For example, lncRNA HNF1A‐AS1 can competitively sponge miR‐93‐5p to enhance CCND1 expression, thereby accelerating the cell cycle progression of CRC cells [362]; lncRNA SNHG12 functions as a sponge for miR‐129‐5p, thereby influencing cell apoptosis and the G1/S phase transition through the upregulation of MAPK1 and E2F7 expression [185]. In LUAD, hsa_circ_0013958 enhances CCND1 expression by sequestering miR‐134, thereby promoting G1 phase progression [363]. In laryngeal squamous cell carcinoma, circ‐CCND1 not only acts as a sponge for miR‐646 but also interacts with the RBP HuR to stabilize CCND1 mRNA, further enhancing its expression [364]. At the signaling pathway level, ncRNAs regulate PI3K/AKT and Wnt/β‐catenin pathways to influence the cell cycle [22]. For example, in HCC, circZFR activates AKT1 signaling by downregulating miR‐511, which leads to the upregulation of CCND1 [365]. In TNBC, circRNA_069718 enhances the expression of its target genes like β‐catenin, which upregulates CCND1 expression and increases cellular proliferation [366].

The rapid division of tumor cells and their self‐protecting response to external stimuli, such as chemotherapy, is due to cell cycle abnormalities. Cancer cells acquire a reversible state of G0 phase arrest termed quiescence. Quiescent cells enhance stem cell properties, such as self‐renewal and multipotency. Furthermore, dormant or quiescent cells have lower metabolic activities. These properties help tumor cells evade chemotherapy‐induced cytotoxicity [367]. Research has shown that ncRNAs have the potential to exert an influence over the quiescent state of CSCs. This is achieved through targeted regulation of cell cycle regulators that are associated with dormancy [368]. For instance, the downregulation of CCND1 mediated by miR‐93/193 and the targeted inhibition of CDK3 by miR‐126 respectively induce GBM cells to enter a quiescent state and develop chemoresistance [369]. Moreover, ncRNAs take part in the regulation of quiescent cancer cell reactivation. In the cancer types of NSCLC and myeloma, the piRNAs piR‐651 and piR‐823 can induce cancer cells to re‐enter the proliferative cycle by promoting the expression of CCND1 and CDK4 [370, 371]. According to a report, regulatory ncRNA networks are promising targets for developing new strategies to effectively deal with cancer and provide patients with modern personalized treatment [368].

ncRNAs establish intricate and dynamic regulatory networks by interacting with factors related to the cell cycle and regulators of cell cycle checkpoints. These results shed new light on how cancer cells escape cell arrest and provide a theoretical basis for cycle‐specific therapies targeting RNA networks. The mechanisms through which noncoding RNAs, or ncRNAs, facilitate the quiescent and subsequent reactive state of cancer cells need further investigation. This is to ensure that these insights help address the clinical challenge of chemoresistance.

### 4.2 Metabolic Reprogramming

The unlimited proliferation of cancer cells relies heavily on a continuous and stable supply of energy and the synthesis of macromolecules. In order to satisfy requirements for sustaining rapid proliferation, cancer cells reprogram their metabolism via multiple pathways, resulting in some striking differences in the metabolic characteristics of the normal cells [372, 373]. Recent studies show that RNA regulatory networks are important for cancer cell metabolic reprogramming. A detailed understanding of these mechanisms should elucidate the principles of tumorigenesis and progression that could aid in generating effective anticancer approaches.

At the posttranscriptional level, various ncRNAs hit metabolic enzymes or signaling pathway key molecules, forming an elaborate regulatory network. lncRNAs have various critical roles in controlling cancer metabolism [374]. Research suggests that lncRNAs that bind to essential TCA cycle metabolic enzymes may impact their protein stability and activity and ultimately cause metabolic reprogramming in tumorigenesis [375]. lncRNAs act as molecular scaffolds that recruit metabolic enzymes, enhancing their interaction with protein or protein complexes for improved function. As an example, lncRNA FEZF1‐AS1 binds to pyruvate kinase 2 (PKM2) specifically and markedly enhances PKM2 protein stability, and as a result, PKM2 levels show a rise in the cytoplasm as well as the nucleus. The interaction stimulates pyruvate kinase activity in colorectal cancer cells, causing aerobic glycolysis [376]. In esophageal squamous cell carcinoma cells, lncRNA AGPG binds to the C‐terminal domain of 6‐phosphofructo‐2‐kinase/fructose‐2,6‐bisphosphatase 3 (PFKFB3), inhibiting ubiquitination at residue Lys302 of PFKFB3. This blockage inhibits proteasome‐dependent destruction, stimulates glycolytic flow, and encourages metabolic reprogramming in tumor cells [377]. On a larger scale, the regulatory networks formed by RBPs and their associated lncRNAs may regulate central metabolic processes. HULC acts as a molecular scaffold that interacts with LDHA and PKM2 and converts them into RNABPs. As a result, the enzymes are more likely to become phosphorylated by fibroblast growth factor receptor 1 (FGFR1), which increases their glycolytic activity and proliferation [378]. In addition, tumor metabolism may be regulated by interactions with other molecules in metabolic signaling pathways. HIF1A‐AS2 promotes the expression of FOXC1 posttranscriptionally by sponging miR‐141‐3p allowing CRC379 to undergo metabolic reprogramming [379].

The epitranscriptomic modifications, especially the m6A modification, play an important role in the regulation of tumor metabolism as well as the expression and function of other metabolism‐related molecules [205]. Methyltransferases, including METTL3 and METTL14, enhance the stability and translation of target mRNAs by directly binding to their respective mRNAs. Studies show that METTL3 can directly activate the pathway of PI3K–Akt–mammalian target of rapamycin (mTOR), which changes the metabolic landscape in cells and allows cancer cells to proliferate, migrate, and invade [380]. Besides, m6A modification controls regulatory ncRNAs that, in turn, influence metabolism. For example, the lncRNA XIST that is regulated by m6A‐methylation can target miR‐126 and affect glucose metabolism levels in glioma cells, which in turn, affects tumor cell proliferation [381].

The glycolytic metabolic pattern of tumors is highly correlated with malignant phenotypes that include tumor proliferation, angiogenesis, lymphangiogenesis, metastasis, and immune escape, which is of important reference value for assessing tumor prognosis and formulating therapeutic strategies [382]. A comprehensive outlook of the role of RNA regulatory networks in the metabolic change of cancer cells can certainly provide new ideas and new targets for tumor therapy. On the one hand, it is possible to develop inhibitors or activators that would target selective RNAs or RNA methylation‐modifying enzymes, thereby reducing the metabolic status of tumor cells. One example would be that the inhibition of METTL3 activity may help reverse the metabolic abnormality of tumor cells and also lower their malignancy. There are also expectations that monitoring expression levels and modification states of key molecules within RNA regulatory networks will aid in the early diagnosis and prognostic evaluation of tumors, ultimately facilitating personalized treatment. The field of RBPs that regulate cellular metabolism—especially in stem cells—would be a very exciting area to investigate further [383]. Future studies should use high‐throughput screening technologies to identify new RBPs in metabolic regulation and develop more sensitive methods to detect potentially important transient or weak RBP–RNA interactions involved in metabolic switching.

### 4.3 Tumor Microenvironment

**FIGURE 4:**
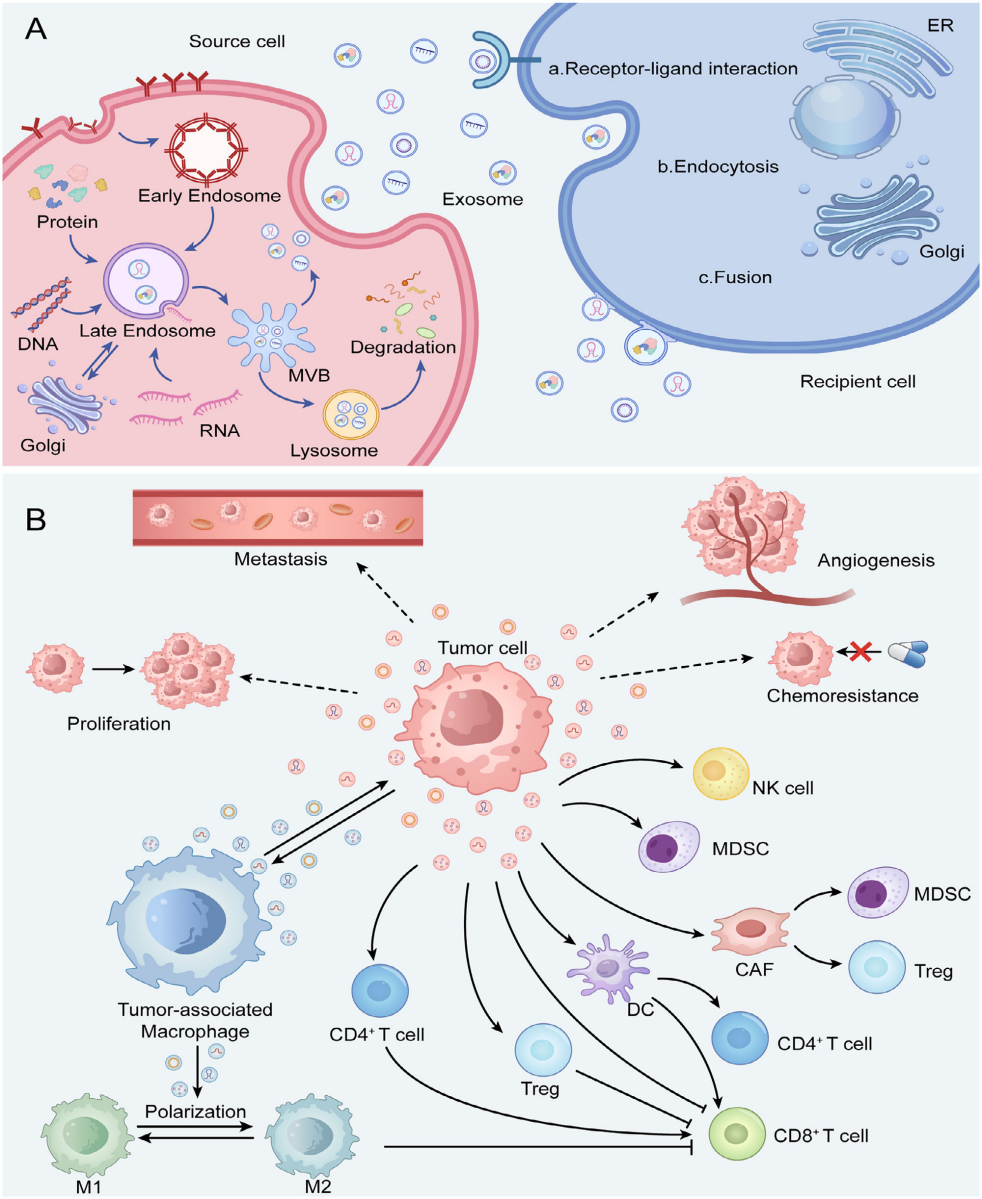
Exosome biogenesis, communication mechanisms, and effects on tumors in the tumor microenvironment. (A) The biogenesis of exosomes commences with the formation of early endosomes through endocytosis. These early endosomes gradually mature into late endosomes via invagination and membrane sorting. The membranes of late endosomes bud inward to generate multiple intraluminal vesicles, ultimately evolving into multivesicular bodies. During this process, biomolecules such as proteins, nucleic acids, and lipids present in the cytoplasm are selectively encapsulated within the intraluminal vesicles. Subsequently, MVBs either fuse with lysosomes for degradation or release exosomes into the extracellular space through exocytosis. The released exosomes convey signals to recipient cells through three primary pathways: target cells phagocytose and internalize exosomes; exosomes fuse with the recipient cell membrane, transferring their contents; and receptors interact with ligands, initiating intracellular cascade reactions. (B) Exosomal ncRNAs within the tumor microenvironment can enhance tumor proliferation, metastasis, angiogenesis, and chemoresistance. Additionally, these ncRNAs contribute to the establishment of an immunosuppressive microenvironment by modulating immune cells.

The TME refers to the environment around the tumor within the tumor stroma. The TME comprises inflammatory cells, immune cells, mesenchymal stem cells, fibroblasts, endothelial cells, pericytes, and the extracellular matrix [384, 385]. The key components of the TME are exosomes, which are 40–150 nm extracellular vesicles. They have a vital role in regulating multiple physiological and pathological processes, especially intercellular communications [386]. Exosomes in the TME can alter the TIME to induce immune suppression that can further assist in tumor growth, beyond their effects on the initiation and growth of tumor cells [387]. RNA is a critical component through which exosomes exert their effects. RNAs within the exosomes can modulate the activities of immune cells, fibroblasts, and other components within the TME, thus playing an essential role in tumorigenesis and progression. Figure 4 demonstrates how exosomes develop and communicate, in addition to the effects that exosomal RNAs have on tumors.

ncRNAs found in exosomes can impact tumor bio‐behaviors as a whole. They can exert their effects by creating interlocking molecular regulatory networks, which hit a key link in tumor initiation and development. The recent research scholar on the role of exosomal ncRNAs playing in the tumorigenesis and development is systematic summarized in Table 3. Via specific binding, modulation of signaling pathways, and molecular trapping, RNA networks upset the natural dial‐operation of oncogenes and tumor suppressor genes in tumor cells, leading to proliferation of tumor cells. As they do this, they also regulate cell cycle and apoptosis‐related molecular mechanisms to further consolidate the malignant proliferative phenotype of tumors. The network remodels the cytoskeleton and activates invasion‐related signaling pathways of tumor cells to enhance their migration and invasion during dissemination. It also modifies how cells in the TME interact with each other in a way that favors tumor metastasis. According to angiogenesis, the RNA networking system can induce neovascularization for supplying essential nutrition and metabolic pathway required for tumor growth via regulating proliferation, differentiation, and functional activity of vascular endothelial cells. The chemoresistance development is closely linked to this network. By regulating drug metabolism, cell survival pathways, and stress responses, it reduces the sensitivity of tumor cells to chemotherapeutic drugs, thereby overcoming a major obstacle to effective clinical treatment [25, 26].

**TABLE 3:** Recent advances in the role of exosomal ncRNAs in tumorigenesis and development.

Biological function	Exosomal ncRNA	Expression level	Potential targets of ncRNA in tumor cells	Mechanism	Cancer types	References
Tumor proliferation	miR‐21	High	PDCD4↓	Promotes tumor progression by targeting and inhibiting PDCD4 expression, thereby preventing apoptosis of GC cells	GC	[388]
	miR‐221‐3p	High	CDKN1B↓	Directly targets and inhibits CDKN1B expression, promoting EOC cell proliferation and G1/S transition	EOC	[389]
	miR‐125a/b	Low	CD90↑	Targets CD90 to promote HCC cell proliferation	HCC	[390]
	miR‐142/223	High	STMN1 and IGF‐1R↓	Downregulates the expression of stathmin‐1 and insulin‐like growth factor‐1 receptor at the posttranscriptional level, inhibiting tumor cell proliferation	HCC	[391]
	miR‐146b‐5p	High	HIPK3↓	Promotes the malignant phenotype of OSCC	OSCC	[392]
	miR‐589‐3p	High	BCL2L13↓	Promotes OC progression through BCL2L13	OC	[393]
	miR‐95	High	*JUNB*↓	Promotes tumor progression by directly binding to its downstream target gene *JUNB*	PCa	[394]
	lncRNA CDKN2B‐AS1	High	P4HA1↑	Exerts oncogenic effects through the miR‐122‐5p/P4HA1 axis	TC	[395]
	lncRNA LIFR‐AS1	High	miR‐29a↓	Acts as a miR‐29a sponge to upregulate NFIA, promoting tumor cell proliferation but inhibiting tumor cell invasion and apoptosis	OS	[396]
	circADRM1	High	MMP14↑	Recruits USP12 to prevent ubiquitination of MMP14 protein, thereby enhancing MMP14 protein stability; MMP14 promotes cell proliferation	LUAD	[397]
	circKDM4A	High	CUL4B↑	Promotes PC malignant progression through the miR‐338‐3p/CUL4B axis	PC	[398]
	circKIAA1797	High	PBX3↑	Regulates GC progression and glycolysis by targeting the miR‐4429/PBX3 pathway	GC	[399]
	circKIF20B	Low	OXPHOS↓	Inhibits gefitinib resistance and cell proliferation by arresting the cell cycle, promoting apoptosis, and reducing OXPHOS through the circKIF20B/miR‐615‐3p/MEF2A axis in NSCLC	NSCLC	[400]
	circHSDL2	High	ZNF704↑	Regulates the Hippo pathway through the miR‐7978/ZNF704 axis, promoting breast cancer malignancy	BC	[401]
	circ‐LMO7	Low	ARHGAP24↑	Exerts tumor‐suppressive effects on OS by sponging miR‐21‐5p and upregulating ARHGAP24 expression	OS	[402]
	circSTAU2	Low	CAPZA1↑	Inhibits GC progression through the miR‐589/CAPZA1 axis	GC	[403]
	circCOL1A2	High	LASP1↑	Sponges miR‐665 to enhance LASP1 expression and regulate CRC phenotypes	CRC	[404]
	circRHOT1	High	PRMT5↑	Regulates the miR‐204‐5p/PRMT5 axis to promote BC progression	BC	[405]
	circ_0001955	High	PGK1↑	Sponges miR‐708‐5p to upregulate PGK1, promoting proliferation and glycolysis	BC	[406]
	circ_0003026	High	XRN2↑	Acts as a molecular sponge for hsa‐miR‐1183 to regulate XRN2 expression, promoting cancer cell growth	NSCLC	[407]
	circ_0006646	High	RRM2↑	Regulates the miR‐758‐3p/RRM2 axis to accelerate cell growth and metastasis	CC	[408]
	circ_001860	High	ZEB1↑	Through competitive binding with miR‐582‐5p, circ_001860 increases ZEB1, thereby promoting in vivo tumor formation.	CRC	[409]
Tumor metastasis	miR‐10527‐5p	High	Rab10↓	Affects Wnt/β‐Catenin signaling through Rab10, thereby inhibiting migration, invasion, lymphangiogenesis, and lymphatic metastasis	ESCC	[410]
	miR‐106a‐5p	High	SOCS6↓	Inhibits SOCS6 and activates the JAK2/STAT3 pathway to promote macrophage M2 polarization; M2 macrophages mutually enhance CRC liver metastasis.	CRC	[411]
	miR‐124‐3p	Low	Rab27a↓	Can directly target the 3′UTR of Rab27a in NSCLC cells, inhibiting exosome secretion. Furthermore, it suppresses the activation of the PI3K/AKT signaling pathway within the intracellular environment, thereby preventing cell migration and invasion.	NSCLC	[412]
	miR‐1911‐5p	High	ZBTB4↓	Promotes migration and invasion of LUAD cells by inhibiting ZBTB4 activated by CELF2	LUAD	[413]
	miR‐21‐5p/miR‐155‐5p	High	BRG1↓	Downregulates BRG1 expression by binding to the BRG1 coding sequence, promoting CRC cell migration and invasion	CRC	[414]
	miR‐200b‐3p	High	ZEB1↓	Downregulation of ZEB1 and upregulation of interleukin‐4 induce M2 polarization. This process activates the JAK/STAT signaling pathway, resulting in the increased expression of PIM1 and VEGFα, which accelerates both proliferation and metastasis.	HCC	[415]
	miR‐92a‐2‐5p	High	AR↓	Targets the 3′UTR of AR mRNA and inhibits AR translation to alter PHLPP/p‐AKT/β‐catenin signaling, promoting HCC cell invasion	HCC	[416]
	miR‐223	High	Mef2c↓	Targets the Mef2c‐β‐catenin pathway to promote BC cell invasion	BC	[417]
	miR‐223	High	PTEN↓	Targets the PTEN–PI3K/AKT pathway, alters the actin cytoskeleton, upregulates multiple EMT‐related proteins, promoting GC cell migration and invasion	GC	[418]
	lncRNA HOXD‐AS1	High	FOXM1↑	Regulates the miR‐361‐5p/FOXM1 axis as a ceRNA, thereby promoting PCa metastasis	PCa	[419]
	LINC00355	High	HDAC3↑	Interacts with HDAC3 to inhibit TP53INP1 transcription, thereby promoting EMT	GC	[420]
	lncRNA Mir100hg	High	miR‐16‐5p and miR‐23a‐3p↓	Promotes tumor cell metastasis by enhancing glycolysis	MEL	[421]
	LncRNA MIR193BHG	High	DNMT3A↑	Targets the miR‐489‐3p/DNMT3A signaling axis in osteoclasts to promote BC bone metastasis	BC	[422]
	lncRNA SNHG3	High	CTNNB1↑	Exosomal SNHG3 is internalized by CRC cells, subsequently upregulating β‐catenin expression by facilitating the nuclear translocation of hnRNPC. This process leads to the activation of EMT and promotes the metastasis of CRC cells.	CRC	[423]
	lncRNA PART1	Low	SOCS6↑	Inhibits OSCC cell viability, migration, and invasiveness through the miR‐17‐5p/SOCS6 axis	OSCC	[424]
	lncRNA AFAP1‐AS1	High	miR‐26a↓	Inhibits miR‐26a as a ceRNA and upregulates ATF2 to promote EC cell migration, invasion, and lung metastasis	EC	[425]
	circATP8A1	High	miR‐1‐3p↓	Induces macrophage M2 polarization and tumor progression through the circATP8A1/miR‐1‐3p/STAT6 axis	GC	[426]
	circCRIM1	High	OGA↑	Promotes TNBC progression by inhibiting miR‐503‐5p and activating the OGA/FBP1 signaling pathway	TNBC	[427]
	circPOLQ	High	miR‐379‐3p↓	Targets miR‐379‐3p to activate the IL‐10/STAT3 axis, thereby promoting M2 macrophage polarization and the formation of metastatic nodules in CRC	CRC	[428]
	circTMCO3	High	ITGA8↑	Promotes malignancy by targeting the miR‐515‐5p/ITGA8 axis	OC	[429]
	circ_0000395	High	MYH9↑	Sequesters miR‐432‐5p to increase MYH9 expression, thereby promoting CRC progression	CRC	[430]
	circ_000200	High	HBEGF↑	Promotes GC progression through the hsa_circ_000200/miR‐4659a/b‐3p/HBEGF axis and affects the expression of TGF‐β/Smad	GC	[431]
	circ_0006906	High	IGF2BP1↑	Regulates the miR‐92a‐1‐5p/IGF2BP1 pathway, promotes PTBP1/IGF2BP1 interaction, and accelerates CRC progression	CRC	[432]
	circ_0020095	High	IRAK1↓	Competitively binds with IGF2BP1, then reduces the binding ability of IGF2BP1 to the IRAK1 3′UTR, thereby inhibiting M1 macrophage polarization and promoting tumor cell progression	COAD	[433]
	circ_0067557	High	Lin28A/B↑	Targets Lin28A and Lin28B to promote the malignant phenotype of CRC cells	CRC	[434]
	circ‐0100519	High	NRF2↑	circ‐0100519 serves as a scaffold that enhances the interaction between USP7 and NRF2 in macrophages. This interaction induces the deubiquitination of NRF2 mediated by USP7 and promotes the polarization of macrophages toward an M2‐like phenotype, thereby facilitating the invasion and metastasis of BC cells.	BC	[435]
Tumor angiogenesis	miR‐421	High	HS2ST1↓	Targets HS2ST1 in vascular endothelial cells to activate angiogenesis	OSCC	[436]
	miR‐501‐3p	High	TGFBR3↓	Targets TGFBR3 to promote angiogenesis	PDAC	[437]
	miR‐1247‐3p	High	FOXO1↓	Inhibits FOXO1 expression and promotes angiogenesis in bladder cancer	BLCA	[438]
	lncRNA MFI2‐AS1	High	NFAT5↑	Increases NFAT5 expression by sponging miR‐107, thereby activating the PI3K/AKT pathway and promoting angiogenesis and migration	NSCLC	[439]
	lncRNA RPPH1	High	IGF2BP2↑	Protects IGF2BP2 from ubiquitination‐induced degradation, stabilizes m6A‐modified FGFR2 mRNA, and activates the PI3K/AKT pathway	BC	[300]
	lncRNA TRPM2‐AS	High	NUMB↓	TRPM2‐AS enhances PABPC1‐mediated inhibition of NUMB expression, ultimately promoting activation of the NOTCH1 signaling pathway and tumor angiogenesis.	GBC	[440]
	circTUBGCP4	High	PDK2↑	Promotes of tip cell formations achieved through the inhibition of miR‐146b‐3p, which activates the Akt signaling pathway. This activation leads to the tilting of vascular endothelial cells, thereby promoting angiogenesis and tumor metastasis.	CRC	[441]
	circNFIX	High	TRIM44↑	Regulates the JAK/STAT1 pathway through the miR‐518a‐3p/TRIM44 axis in HUVECs, thereby promoting angiogenesis	OC	[442]
	circCOL1A1	High	EIF4A3↑	Recruits EIF4A3 and activates Smad2/3 signaling to promote angiogenesis	CRC	[443]
	circTUBGCP4	High	PDK2↑	Promotes tip cell formation by inhibiting miR‐146b‐3p and activates the AKT signaling pathway	CRC	[441]
Tumor chemoresistance	miR‐130a‐3p	High	FSP1↓	Regulates ferroptosis by inhibiting METTL14‐mediated m6A RNA methylation of FSP1, thereby conferring DDP resistance	EC	[262]
	miR‐20	High	RB1↓	Targets the 3′UTR of RB1 mRNA and negatively regulates RB1 expression to reduce cell sensitivity to CDK4/6i	BC	[444]
	miR‐200b‐3p	Low	ZEB1 and E2F3↑	Loss of exosomal miR‐200b‐3p may lead to CRC progression and reduced sensitivity to 5‐Fluorouracil by upregulating ZEB1 and E2F3.	CRC	[445]
	miR‐181b‐5p	High	p53/p21↓, BCLAF1↓	Overexpression of miR‐181b‐5p downregulates p53 and p21 levels, inhibiting adriamycin‐induced G1 phase arrest and senescence by suppressing BCLAF1 expression in vitro.	BC	[446]
	miR‐21	High	PTEN↓	Activates PI3K/AKT signaling by downregulating PTEN to inhibit GC cell apoptosis and promote DDP resistance	GC	[447]
	miR‐3662	High	RBMS3↓	Promotes cellular processes and gemcitabine resistance by targeting RBMS3	BC	[448]
	miR‐3681‐3p	High	MLH1↓	Targets MLH1 to induce DDP resistance in gastric cancer cells	GC	[449]
	miR‐21	High	STAT3 and PDCD4↓	Regulates the STAT3/miR‐21/PDCD4 signaling pathway, which is associated with temozolomide resistance in GBM	GBM	[450]
	miR‐223	High	PTEN↓	Activates downstream signaling pathways, including PI3K/AKT, by downregulating PTEN to enhance drug resistance of tumor cells under hypoxia	PDAC	[451]
	lncRNA CRNDE	High	PTEN↓	Promotes NEDD4‐1‐mediated PTEN ubiquitination to increase DDP resistance in GC cells	GC	[452]
	lncRNA SNHG5	High	ATG5↑	Induces a CAFs‐like phenotype and autophagy in AML–mesenchymal stem cells through the PTBP1/ATG5 axis, which enhances the stability of ATG5 mRNA, thereby conferring chemoresistance to AML cells	AML	[453]
	lncRNA DACT3‐AS1	Low	SIRT1↑	Inhibits cell proliferation, migration, and invasion by targeting the miR‐181a‐5p/SIRT1 axis. DACT3‐AS1 enhances the sensitivity of cancer cells to oxaliplatin through SIRT1‐mediated ferroptosis both in vitro and in vivo.	GC	[454]
	lncRNA FAL1	High	Beclin1↓	Acts as a scaffold for the interaction between Beclin1 and TRIM3, promoting the TRIM3‐dependent polyubiquitination and degradation of Beclin1, thereby inhibiting oxaliplatin‐induced autophagic cell death	CRC	[455]
	lncRNA ZNF667‐AS1	Low	TGFBR1↓	ZNF667‐AS1 interacts with U2AF1, disrupting the stability of TGFBR1 mRNA in CD4+ T cells and reducing TGFBR1 expression.	PC	[456]
	Linc00852	High	COMMD7↑	Targets miR‐514a‐5p to regulate COMMD7 expression, rendering cells resistant to DDP	GC	[457]
	circ_0010467	High	LIF↓	Mediates DDP resistance through the AUF1/hsa_circ_0010467/miR‐637/LIF/STAT3 axis	OC	[458]
	circ_0076305	High	ABCC1↑	Enhances ABCC1 expression by sponging miR‐186‐5p, thereby regulating DDP resistance in NSCLC	NSCLC	[459]
	circSYT15	High	RSF1↑	Promotes DDP resistance in cervical cancer cells through the miR‐503‐5p/RSF1 axis	CC	[460]
	circPARD3	High	SIRT1 and SSRP1↑	Promotes EBV‐miR‐BART4‐induced stemness and DDP resistance in NPC‐SP cells through the miR‐579‐3p/SIRT1/SSRP1 axis	NPC‐SP	[461]
	circKIF20B	Low	OXPHOS↓	Inhibits gefitinib resistance and cell proliferation by arresting the cell cycle, promoting apoptosis, and reducing OXPHOS through the circKIF20B/miR‐615‐3p/MEF2A axis in NSCLC	NSCLC	[400]
Immunosuppression/escape	miR‐372‐5p	High	PD‐L1↓	Regulates PD‐L1 expression in colorectal cancer cells and macrophages by targeting the PTEN/AKT/NF‐κB pathway, which induces an immunosuppressive microenvironment in CRC and promotes the development of CRC	CRC	[462]
	lncRNA NEAT1	High	PBX1↓	Regulates the EZH2/PBX1 axis to inhibit NK cell activity, thereby promoting immune escape of multiple myeloma cells	MM	[463]
	circ_001264	High	RAF1↑	Regulation of RAF1 expression activates the p38–STAT3 signaling pathway, which thereby induces M2 macrophage polarization. Polarized M2 macrophages can subsequently induce PD‐L1 overexpression, thereby promoting immunosuppression in AML.	AML	[464]
	circRNA_0013936	High	FATP2↑ and RIPK3↓	Upregulates FATP2 through the circRNA_0013936/miR‐320a/JAK2 pathway and downregulates RIPK3 in PMN‐MDSCs via the circRNA_0013936/miR‐301b/CREB1 pathway, leading to a significant inhibition of CD8+ T cell function	BLCA	[465]

Abbreviations: AKT, protein kinase B; BC, breast cancer; BLCA, bladder cancer; CC, cervical cancer; COAD, colon adenocarcinoma; CRC, colorectal cancer; DDP, cisplatin; EC, esophageal carcinoma; EOC, epithelial ovarian cancer; ESCC, esophageal squamous cell carcinoma; FOXM1, Forkhead Box M1; GBC, gallbladder cancer; GC, gastric cancer; HCC, hepatocellular carcinoma; IL, interleukin; LUAD, lung adenocarcinoma; MEL, melanoma; MM: multiple myeloma; MMP14, matrix metallopeptidase 14; NPC‐SP, nasopharyngeal carcinoma side population; NSCLC, non‐small cell lung cancer; OC, ovarian cancer; OS, osteosarcoma; OSCC, oral squamous cell carcinoma; PC, pancreatic cancer; PCa, prostate cancer; PDAC, pancreatic ductal adenocarcinoma; PI3K, phosphatidylinositol 3‐kinase; PTEN, phosphatase and tensin homolog; SOCS6, suppressor of cytokine signaling 6; STAT3, signal transducer and activator of transcription 3; TGF‐β, transforming growth factor‐beta; TP53INP1, TP53 inducible nuclear protein 1; USP12, ubiquitin specific peptidase 12; VEGFα, vascular endothelial growth factor alpha; ZEB1, zinc finger E‐box binding homeobox 1.

Within the TME, both tumor and nontumor cells, such as fibroblasts and immune cells can secrete exosomes that collectively shape an immunosuppressive microenvironment [466]. The TIME consists of distinct immune cell populations and their interactions within the TME niche, which has garnered significant attention due to its critical role in carcinogenesis, cancer progression, and therapeutic responses [467]. Exosomal ncRNAs reshape the TIME by regulating the functions, interactions, and infiltration of immune cells, fibroblasts, and myeloid‐derived suppressor cells [25]. The exosomal ncRNAs have been shown to play a dual role in immune evasion by tumors, whereby some of the tumor‐derived exosomal ncRNAs such as miRNAs are able to inhibit dendritic cell (DC) function, natural killer (NK) cell and CD8+ T cell cytotoxic activity, and promote M2 macrophage polarization and regulatory T cell (Tregs) immunosuppressive function to help escape immune surveillance by tumors. Conversely, other tumor‐derived exosomal ncRNAs enhance DC function, inhibit M2 macrophage polarization, and improve CD8+ T cell cytotoxicity, thereby strengthening antitumor immune responses [468]. Macrophages abundant in the TME, exist in two polarized phenotypes: M1 and M2 [469]. The polarization toward the M2 phenotype promotes tumor progression and mediates therapeutic resistance [470]. For instance, in PCa, exosome‐enriched miRNAs such as let‐7a‐5p, let‐7b, let‐7 g, and let‐7i downregulate integrin‐β3, thereby inducing M2 polarization [471]. In HCC cells, the highly expressed exosomal lncRNA TUC339 promotes M2 polarization, which leads to a reduction in proinflammatory cytokine production, impaired phagocytosis, and downregulation of costimulatory molecule expression [472]. Conversely, miR‐21 in CRC exosomes promotes M1 polarization via toll‐like receptor 7, inducing interleukin‐6 (IL‐6) production, which in turn creates a proinflammatory premetastatic niche that facilitates CRC cell survival, colonization, and liver metastasis [473].

Hypoxia, elevated lactate levels, extracellular acidosis, and limited nutrient availability are characteristic features of the TME [474, 475]. Stressors such as hypoxia, nutrient deprivation, and acidosis enhance the release of exosomes by malignant cells, thereby fostering the dynamic evolution and expansion of the TME and driving tumor progression [476]. Hypoxic circumstances lead to glioma stem cell exosomes high in IL‐6 and miRNA‐155‐3p that activated the signal transducer and activator of transcription (STAT)3 pathway, which in turn highlights IL‐6 release. The expression of miRNA‐155‐3p, which was upregulated by IL‐6, enhances glioma development via autophagy induction and M2 macrophage polarization [477]. An analogous process occurs when CRC cells secrete exosomes rich in miRNA‐210‐3p, which subsequently causes downregulation of *CELF2*. This ultimately inhibits apoptosis and drives the G1‐to‐S phase transition. Research on cancer exosomes has shown that miRNA‐210‐3p levels are significantly elevated in CRC patients. In CRC patients, miRNA‐210‐3p levels are closely linked with the poor prognosis [478]. Notably, RNA and RBPs also play a crucial role in regulating the uptake and secretion of exosomes [479, 480].

Exosomal RNAs play a crucial role in shaping the immunosuppressive and prometastatic characteristics of the TME by regulating intercellular signaling, immune cell functions, and metabolic stress responses. The bidirectional effects of exosomal RNAs warrant that their clinical application should be strictly differentiated according to their origin and function. Creating RNA delivery systems that can specifically target the TME may enhance immunotherapy options. Right now, modified exosomes that have surface decorations and internal therapeutic molecules (i.e., engineered exosomes) can deliver antitumor drugs to tumor sites with high efficiency and precision while inducing fewer treatment side effects. However, the clinical translation of engineered exosomes has many obstacles to overcome.

### 4.4 Invasion and Metastasis

Epithelial–mesenchymal transition (EMT) is a crucial biological process during normal embryonic development and in tissue regeneration that is essential for the maintenance of homeostasis of the organism. The abnormal activation of the EMT enables tumor cells to acquire multiple malignant traits, including higher migratory and invasive capacities, enhanced CSC properties, and greater resistance to chemotherapy and immunotherapy during cancer progression and metastasis [481]. This process is tightly regulated by a complex network of intrinsic and extrinsic factors, which include transcription factors (e.g., SNAIL, ZEB, TWIST), posttranslational modifications, epigenetic modifications, and precise regulatory mechanisms mediated by ncRNAs [482, 483].

ncRNAs primarily regulate cancer cell metastasis and invasion by influencing EMT transcription factors or related structural proteins. A typical regulatory model is observed in the miR‐200 family, which includes miR‐200a, miR‐200b, and miR‐200c, and is downregulated in various tumor tissues. These miRNAs directly bind to the mRNAs of *ZEB1/ZEB2* mRNAs to inhibit the expression of these transcription factors. This action This action relieves the repression of epithelial genes by ZEB proteins, effectively suppressing the EMT process [484]. Interestingly, ZEB1 and ZEB2 can bind to regulatory elements in the miR‐200 promoter, inhibiting miR‐200 transcription and forming a negative regulatory feedback loop that promotes EMT [485]. In NSCLC tissues, significantly upregulated miR‐196b‐5p promotes lung cancer cell migration by directly targeting the tumor suppressors GATA6 and TSPAN12 [486]. Beyond miRNAs, other ncRNAs exhibit unique regulatory mechanisms: for instance, lncRNA H19 has been reported to act as the sponge of miR‐22‐3p and relieve the inhibition of matrix metalloproteinase 14 induced by miR‐22‐3p, culminating in lung metastasis in CRC487 scrutiny [487]. In circRNA research, circITGB6 and circ‐IARS facilitate tumor metastasis and progression. Presented in a TGFβ‐driven EMT context, circITGB6 promotes tumor metastasis in vivo by stabilizing *IGF2BP3* mRNA. circ‐IARS enhances vascular permeability and, thus, tumor invasiveness through the miR‐122–ZO‐1 axis and the RhoA pathway [488, 489].

Apart from RNAs, RBPs that bind to RNAs are also crucial in cancer metastasis [490]. Changes in RBP function is correlated with the development of many human ailments, especially cancer [490]. RBPs are important factors that participate in RNA biogenesis. For example, KH‐type splicing regulatory protein (KSRP), a multifunctional RBP, has been shown to promote the maturation of special microRNAs by interacting with their various precursor microRNAs. Research has shown that KSRP is involved in TGFβ‐mediated EMT by facilitating the maturation of miR‐192‐5p, which targets multiple EMT‐related factors [491]; in addition, KSRP promotes the maturation of miR‐23a, which downregulates early growth response protein 3 expression by directly targeting its 3′UTR, and thus effectively inhibits the invasion and metastasis of NSCLC cells [492]. RBPs may act as oncogenes or tumor suppressors by regulating the stability and processing of lncRNAs and circRNAs [490]. At the posttranscriptional stage, the alternative splicing of mRNA molecules, mediated by RBPs, is an important regulatory mechanism contributing to mRNA stability. The splicing factor quaking (QKI) is an important regulator of circRNA formation during EMT in humans [493]. In PCa cells, the circZEB1 expression is upregulated by QKI. Subsequently, circZEB1 enhances the protein expression of mesenchymal‐related transcription factor ZEB1 via competing for the binding to miR‐141‐3p [494]. Additionally, the alternative splicing of *CD44* mRNA is coordinately regulated by multiple RBPs, and its aberrant splicing is closely associated with EMT progression and cancer metastasis [495]. In studies of lung cancer mutant MORC2 interacts with heterogeneous nuclear ribonucleoprotein M to promote shifts in the alternative splicing patterns of CD44, thereby driving the invasion and metastasis of TNBC cells [496]. RBPs are also known to regulate RNA stability, intracellular localization, translation efficiency, and RNA modifications through various means, which altogether regulates the entire EMT process [490].

EMT in tumors represents a range of intermediate states instead of a binary process [497]. The role of EMT in cancer is somewhat controversial and contradictory, according to existing studies. The primary discrepancies are thought to arise from differences in tissue, model limitations as well as the complexity of cell states [482]. First, some studies reveal that loss of EMT does not prevent metastasis in some models. This indicates the existence of functional redundancy or alternative mechanisms [498]. Second, when we refer to the connection between EMT and tumor stemness, it was generally accepted that the mesenchymal epithelial transition boosted stem cell qualities. More recently, it was shown that a fully mesenchymal state could reduce stemness. However, tumors with a partial EMT state, or intermediate states, have a greater invasive capability [499]. Third, “complete EMT” induced in vitro is rare in vivo; animal models often overlook the influence of the immune microenvironment, and detection methods relying on single biomarkers struggle to accurately capture the spectral characteristics of EMT [500, 501]. The existing contradictions point to the need to examine EMT with respect to tumor type and stage and also the microenvironment. In addition, models and detection methods should be optimized so as to accurately reveal the biological significance of EMT in cancer [482].

### 4.5 Angiogenesis

The angiogenesis of a tumor enables distant metastasis of solid tumors as well as their growth. RNA regulatory networks play an important role in regulating angiogenesis by inducing posttranscriptional modifications and mediating exosomal communication between cells. Besides, they have a vital role in tumorigenesis.

RNA is a fundamental component of the regulatory network governs tumor angiogenesis. ncRNAs primarily influence angiogenesis by modulating the stability and translation of angiogenesis‐related mRNAs. For example, the miR‐200 family effectively inhibit angiogenesis by directly targeting IL‐8 and the chemokine CXCL1 secreted by tumor endothelial cells and cancer cells, or by indirectly regulating associated signaling pathways [502]. Conversely, under hypoxic conditions, HIF‐1α activates the expression of miR‐210, which promotes neovascularization by enhancing the activity of the vascular endothelial growth factor (VEGF) signaling pathway [503]. lncRNAs such as H19 and MALAT1 can upregulate VEGF expression and drive angiogenesis by competitively binding to miRNAs or activating the STAT3 signaling pathway [504, 505]. circRNAs like circDENND4C indirectly activate pathways such as VEGFR2 by sequestering miRNAs. Furthermore, the critical role of the piRNA family member piR‐31115 in regulating tumor angiogenesis has been increasingly confirmed [506]. In addition to ncRNAs, the regulation of mRNA stability (e.g., *VEGF* mRNA regulated by the RBP HuR) and translation efficiency (e.g., significantly enhanced translation efficiency of *HIF‐1α* mRNA under hypoxia) also directly impacts the expression of angiogenesis‐related genes [507, 508]. Interestingly, the secretion of extracellular exosomes is significantly increased in response to the hypoxic TME. This has led to considerable attention on the role of miRNAs carried by tumor‐derived exosomes in regulating angiogenesis [509]. Studies have confirmed that exosomal miR‐21 activates STAT3, which, in turn, elevates VEGF levels in recipient cells, thereby inducing angiogenesis and malignant transformation in human bronchial epithelial cells [510]. Also, exosomal miR‐25‐3p regulates the expression of VEGFR2, ZO‐1, occludin, and Claudin5 in endothelial cells targeting KLF2 and KLF4, further highlighting the diverse roles of exosomal miRNAs in the regulation of tumor angiogenesis [511].

RNA modifications (e.g., m6A) play a central role in tumor angiogenesis by dynamically regulating key signaling pathways and effector molecules [512]. Methyltransferases METTL3/METTL14 directly enhance the expression of proangiogenic factors such as the VEGF and CTGF via modification of the TGFβ and HIF‐1α mRNAs. Moreover, they enhance the hypoxic stimulation of TGFβ1 signaling, leading to EMT and neovascularization [513, 514]. Conversely, FTO inhibits the antiangiogenic chemokine CCL19 by reducing mRNA methylation levels, resulting in increased microvessel density and exacerbated tumor angiogenesis [515]. The biological processes and functions of these agents can interact to form an epigenetic regulatory network that promotes angiogenesis and remodelling of the TME, which in turn promotes cancer progression and metastasis.

Tumor angiogenesis is accurately regulated by RNA regulatory networks in various dimensions, such as regulation of gene expression through modification, miRNA signaling mediated by exosome transport, and synergistic effect of ncRNA on mRNA. A detailed study of the molecular players within RNA regulatory networks will lay the groundwork for cancer treatment strategies targeting angiogenesis, as well as provide novel therapeutic targets.

## 5 RNA Regulatory Networks and Tumor Heterogeneity

Tumor heterogeneity exists in malignant tumors and is characterized by, differences in the growth rate and invasiveness of tumors as well as differences in drug sensitivities and prognosis. It is caused due to the large number of genetic and molecular changes that occur in tumor cells due to their incessant divisions. Tumor heterogeneity causes a multitude of different genotypes and phenotypes to exist within tumors. Such diversity creates subclonal cell populations with their own biological agendas. There are two types of tumor heterogeneity. The first one is spatial heterogeneity, which means the changes between different regions of the same tumor. The second one is temporal heterogeneity. It refers to the differences between the primary and secondary tumors. Both types of heterogeneity have a profound impact on tumor evolution and therapy efficacy.

### 5.1 Tumor Heterogeneity and RNA Expression Profiles

There are two types of tumor heterogeneity, including inter and intra. Genetic and phenotypic variations between different tumor cells categorize into intertumoral heterogeneity, whereas the genetic and phenotypic variations between cells of the same tumor involves intratumoral heterogeneity. This section primarily addresses intratumor heterogeneity. Tumors are often heterogeneous. One way this effect manifests is with the variation in the RNA expression profile of different subpopulations of cells within the same tumor tissue, this includes both the protein‐coding mRNAs and the ncRNAs. The spatial distribution patterns and molecular regulation mechanism exhibit multidimensional complexities.

Evidence from different lines shows that RNA expression is regionally specific within the tumor studied. For example, GBM is marked by large differences in miRNA expression throughout core, margin and invasive edge. In particular, when comparing the invasive edge with both the core and margin, miR‐330‐5p and miR‐215‐5p were upregulated, and miR‐619‐5p, miR‐4440, and miR‐4793‐3p were downregulated [516]. The expression levels of miRNA between the specimen taken from the boundary of the tumor and the intratumor regions are significantly different in BC. Eleven miRNAs, including miRNA‐20a, ‐21, and ‐125b, demonstrate significant differences between tumor boundary and central specimens (*p* < 0.05). Additionally, eight miRNAs, such as miRNA‐126, ‐221, and ‐222, show differential expression between tumor boundary and tumor P1 specimens (*p* < 0.05). Furthermore, miRNAs like miRNA‐145 and ‐451a exhibit dynamic changes across subregions (e.g., P1/P2) [27]. In HCC, miRNA analysis of different compartments within liver tumors indicates that miRNA dysregulation varies across tumor compartments 186 [517].

RNA regulatory networks drive cancer heterogeneity through multidimensional crosstalk. For instance, in pancreatic cancer (PC), H19 can also regulate the expression of the m6A reader protein YTHDC1 by competitively binding to miR‐107. The two synergistically stabilize the splicing factor SRSF1, thereby regulating the alternative splicing of IL‐6 and IL‐10, promoting the polarization of tumor‐associated macrophages toward the M2 phenotype, and exacerbating cancer heterogeneity [518]. BC subtypes and stages show differential expression patterns of m6A enzymes, which can serve as their diagnostic markers [519]. Epigenetic control also contributes to this heterogeneity, such that immune phenotypes can be distinguished by differences in DNA methylation status of lncRNAs in gliomas. Patients displaying hypomethylated cluster C1 (includes GBM and LGG) have a poorer prognosis, and patients with hypermethylated cluster C2 (Largely LGG) have a better prognosis, indicating a correlation between methylation levels and malignancy [520].

The effective management of cancer treatment is hugely dependent on the detection of relevant molecular subtypes or therapeutic targets. Despite achieving great progress in terms of personalized therapy under tumor heterogeneity, the very rapid emergence of adjuvant, neoadjuvant, and metastatic tumor‐targeted drugs emphasize the significant clinical challenge in optimizing precision therapy to enhance patient survival and minimize late recurrence.

### 5.2 RNA Regulatory Networks and CSC Properties

Another important aspect of tumor heterogeneity is represented by CSCs. Research shows us that although a tumor is made up of a lot of tumor cells, only some of these cells can initiate or sustain the tumor. These cells are referred to as CSCs or tumor‐initiating cells [521]. A comprehensive understanding of the biological characteristics of CSCs can help in understanding how cancers originate and evolve and can also enhance therapies.

RNA regulatory networks play an important role in regulating the maintenance and differentiation of CSCs. ncRNAs maintain stem cell properties of cancer cells through signaling pathways [28]. For example, the anomalous activation of the Wnt/β‐catenin signalling pathway is an important contributor to the tumorigenesis and differentiation driven by CSCs. In NSCLC, the protein kinase membrane‐associated tyrosine/threonine 1 (PKMYT1)‐related lncRNA sponges miR‐485‐5p to upregulate PKMYT1, which inhibits β‐transducin repeat‐containing protein 1 (β‐TrCP1)‐mediated degradation of β‐catenin, thereby activating the WNT signaling pathway [522]. Similarly, in liver CSCs, lncSNHG5 activates the Wnt/β‐catenin pathway by inhibiting upstream frameshift 1, thereby maintaining cellular stemness [523]. In other pathways, lncRNA ASB16 antisense RNA 1 (ASB16‐AS1) collaborates with ATM kinase to phosphorylate tripartite motif‐containing 37 (TRIM37), thereby activating the nuclear factor kappa‐B (NF‐κB) pathway and promoting stemness in GC cells [524]. Additionally, the Let‐7a/rat sarcoma (Ras)/NF‐κB axis serves as a stemness‐antagonizing pathway in breast CSCs, where Let‐7a inactivates the NF‐κB pathway in a Ras‐dependent manner [525]. Conversely, some RNA molecules act to inhibit CSC stemness; for example, the miR‐139/phosphodiesterase 2 (PDE2A)/Notch1 regulatory loop in GSCs diminishes stemness and tumorigenicity by suppressing the WNT signaling pathway [526]. Furthermore, miR‐34a is downregulated in GBM cells and inhibits cell growth by targeting Notch1 [527].

RNA editing, plays a crucial role in the regulation of gene expression and is significantly involved in the biological processes of CSCs [528]. miRNAs can modulate the self‐renewal properties of CSCs by targeting genes associated with stemness. Specifically, miR‐17 targets ADAR2, an editing enzyme essential for sustaining the stemness of melanoma stem cells. As melanoma stem cells have reduced expression of miR‐17, which functions as a tumor suppressor by restricting stemness while promoting their differentiation [528]. Furthermore, regulatory mechanisms involving m6A modification are equally vital. In GSCs, METTL3‐mediated m6A modification leads to the overexpression of LINC00839, which enhances c‐Src‐driven β‐catenin phosphorylation, thereby activating WNT signaling and promoting the maintenance of stemness [529]. In CRC, METTL3‐mediated m6A modification induces Sec62 expression, facilitating the nuclear translocation of β‐catenin and reducing its ubiquitin‐dependent degradation, which ultimately sustains cancer stemness [530].

RNA editing, ncRNAs, and epigenetic modifications establish networks that maintain CSC stemness at several levels, either by modifying posttranslational modifications or targeting relevant signalling pathways. These factors have become prominent players in the development of tumor therapeutic resistance. Nevertheless, at the present time, most studies are single pathway or single molecule. It is essential to construct dynamic regulatory network models and validate combination strategies targeting CSCs by using organoid and single‐cell technologies in future studies.

## 6 RNA Regulatory Networks in Cancer Diagnosis and Prognosis

The RNA expression profiles hold a highly significant value in cancer and cancer biomedical research. The mechanistic level helps us unravel the molecular mechanisms of tumorigenesis; discover new oncogenes, tumor suppressor genes and biomarkers; and better understand the heterogeneity of tumors. The RNA expression profile can have applications in the clinic to discover early diagnostic markers, define tumor types, and assess prognosis. In therapy, they are useful in making strategies for individuals, checking therapy responses and finding new targets to treat cancer, leading toward precision medicine [531, 532].

### 6.1 RNA Biomarkers in Liquid Biopsies

The body fluid (e.g., blood, urine, feces, saliva)‐based testing (liquid biopsy) to analyze tumor‐derived substances (circulating tumor cells, circulating tumor DNA, circulating tumor RNA, platelets, exosome etc.) is an emerging pathological detection technology. The application generates vital information to facilitate early diagnosis, disease state measurement, therapeutic monitoring, and prognosis in tumoral patients. Due to its noninvasiveness and convenience, liquid biopsy has a very broad application prospect in clinical practice [533, 534].

Circulating miRNAs have been found to be very stable in different biofluids (including blood, plasma, serum, and saliva) and are resistant to degradation by endogenous RNases, making them great biomarkers [30]. Research shows that panel of different miRNAs (miR‐1246, miR‐1307‐3p, miR‐4634, miR‐6861‐5p, and miR‐6875‐5p) can be used for BC detection where identification of specific miRNA panels helps in identifying cancer as well as early‐stage patients [535]. The analysis of circulating miRNA profiles in bodily fluids can effectively separate patients from healthy persons. Urine sample analysis shows good reliability, reproducibility, and stability [536, 537]. In addition, aberrantly expressed miRNAs in feces and serum show significance as cancer diagnostic markers. The use of blood miRNAs for the diagnosis of CRC shows high sensitivity but low specificity. However, the simultaneous detection of multiple miRNAs can produce a much‐improved diagnostic accuracy relative to single‐miRNA detection [538]. A number of lncRNAs or their processed fragments were stable in human body fluids and could be detected in plasma and urine from cancer patients. Their expression levels correlate with disease severity, thus positioning them as potential tumor biomarkers. Research has shown that exosomal H19 expression in the serum of BC patients is significantly increased compared with patients without malignancy and exhibits better diagnostic value than standard markers [539]. Urinary PCA3 expression positively correlates with disease severity, and is significantly upregulated in PCa [540]. mRNA and protein biomarkers have emerged as potential early biomarkers of BC, being detected in saliva to differentiate between patients and healthy individuals [541].

Exosomes are advantageous materials for liquid biopsy as they display high stability and can comprehensively reflect tumor cells’ characteristics. Useful for the diagnosis and treatment of cancer [542]. For example, the upregulation of exosomal miR‐1246, miR‐4644, miR‐3976, and miR‐4306 can be highly sensitive biomarkers for PCa patients [543]; similarly, the exosomal lncRNA H19 expression is elevated in the serum of BLCA patients, which suggests that exosomal lncRNAs may be important diagnostic markers [544]. Also, cancer type separation can be performed with specific miRNA‐containing exosomes. Plasma exosomes enriched with miR‐30e‐3p, miR‐30a‐3p, miR‐181‐5p, and miR‐361‐5p serve as specific diagnostic biomarkers for adenocarcinoma while plasma exosomes enriched with miR‐15b‐5p, miR‐10b‐5p, and miR‐320b serve as specific diagnostic biomarkers for squamous cell carcinoma [31]. The exosomal miRNAs have potent capabilities to work as inhibitors of tumor progression in cancer therapy, in addition to tumor markers. As an illustration of the above, the transfer of miR‐142 and miR‐223 influences the posttranscriptional regulation of proteins in HCC. This transfer reduces the expression of reporter proteins and endogenously expressed stathmin‐1 and insulin‐like growth factor‐1 receptor, inhibiting the proliferation of cancer cells. Thus, miR‐142 and miR‐223 may serve as inhibitors in tumor therapy [391].

Nevertheless, to use exosomes as biomarkers, it is required to solve technical obstacles, attain technical standardization, optimize detection sensitivity, as well as specificity. Every exosome isolation technique has certain benefits and drawbacks. The specific need of the research, like purity, throughput, resources, and so on, determines the choice of technology [545]. The problem is: there is a lack of standardization in the protocols used for exosome isolation and quantification. This makes the reproducibility and reliability of biomarker detection derived from exosomes problematic and hinders the translation [546]. Addressing this challenge requires sustained research and development efforts, investment in next‐generation isolation and analysis technologies, and the establishment of globally recognized standards. Second, exosomes present in body fluids are heterogeneous and originate from different types of tissues. Therefore, it is hard to distinguish tumor signals from “noise” of normal cells, in RNA transcriptomic analysis. At present, exosomes can be isolated according to tissue origin using the capture of specific exosomal surface proteins with immunoprecipitation and nanoflow cytometry. These technologies can enhance particular exosomes and amplify disease indications, which can enhance the performance of exosome‐based RNA analysis [547]. Third, to improve diagnostic and prognostic accuracy, future studies should focus not only on mRNA or ncRNA but also on multiple exosomal cargos (mRNA, ncRNA, proteins, metabolites) [548]. In fact, due to the unique vesicular structure of the exosomes multianalyte testing is suitable. Combined biomarker panels that integrate exosomal RNA with other exosomal cargos reflecting different aspects of exosomes will provide precise and multidimensional information for cancer diagnosis, prognosis, and prediction [549].

Liquid biopsy is an important breakthrough as it enables early screening, diagnosis, and treatment of cancer where tissue samples are not available. The transient molecular, genetic, and epigenetic features of tumor cells, identified from earlier therapies as drug‐resistant clones, can be assessed using circulating biomarkers obtained from liquid biopsy. These noninvasive tools make it easy to track responses to ongoing treatments. The data from these tools help guide rapid medical decision making regarding the next cycles of chemotherapy or if the plan needs to be amended or changed [550]. Table 4 presents circulating RNAs as potential biomarkers in liquid biopsies. Circulating RNAs can be considered as biomarkers in liquid biopsies. Still, the sensitive detection of RNA remains challenging in liquid biopsies as RNA is low in frequency, unstable, and the absence of standardized protocols between the different platforms such as quantitative reverse transcription‐polymerase chain reaction, microarrays, and next‐generation sequencing [551]. It is necessary to develop more sensitive and specific biomarkers and to generate more evidence‐based medical data for liquid biopsy to move from research to clinical practice. We must begin to develop better and more scalable protocols for the separation of circulating RNAs (especially exosomal RNAs) using advanced molecular biology detection technologies that increase the specificity and reliability of liquid biopsies [552]. In near future, liquid biopsy is expected to be crucial in early diagnosis, targeted treatment, ongoing monitoring, and prognostic assessment of cancer.

**TABLE 4:** Circulating RNA biomarkers in liquid biopsies.

Cancer type	RNA types	RNA	Sample	Expression level	Function	References
BC	miRNA	miR‐21, miR‐125b, miR‐451	Urine	↓	Diagnosis	[553]
		miR‐16, miR‐21, miR‐451	Plasma	↑	Diagnosis and prognosis prediction	[554]
		miR‐145	Plasma	↓	Diagnosis and prognosis prediction	[554]
		miR‐155	Serum, urine	↑	Diagnosis	[553, 555]
		miR‐205	Serum	↓	Diagnosis	[554]
		miR‐301a‐3p	Blood	↑	Diagnosis and prognosis prediction	[556]
	lncRNA	LINC00511	Blood	↑	Diagnosis and prognosis prediction	[556]
		lncRNA H19	Serum exosomes	↑	Diagnosis and prognosis prediction	[539]
CRC	mRNA	KRTAP5‐4, MAGEA3	Serum exosomes	↑	Diagnosis	[557]
	miRNA	miR‐221, miR‐18a	Feces	↑	Diagnosis	[558]
		miR‐223, miR‐451	Feces	↑	Diagnosis	[559]
		miR‐21, miR‐92a	Feces	↑	Diagnosis	[560]
		miR‐106a	Feces	↑	Diagnosis	[561]
		miR‐135b	Feces	↑	Diagnosis	[562]
		miR‐144	Feces	↑	Diagnosis	[563]
		miR‐143, miR‐145	Feces	↓	Diagnosis	[564]
		miR‐4478, miR‐1295b‐3p	Feces	↓	Diagnosis	[565]
		miR‐92b	Plasma	↑	Diagnosis	[566]
	lncRNA	HOTTIP	Serum exosomes	↑	Diagnosis and prognosis prediction	[567]
		BCAR4	Serum exosomes	↑	Diagnosis	[557]
	circRNA	circLPAR1	Serum exosomes	↑	Diagnosis	[568]
		circ‐PNN	Serum exosomes	↑	Diagnosis	[569]
GC	mRNA	KAT2B	Plasma	↓	Diagnosis	[570]
		MAOB‐NAB2	Saliva	↑	Diagnosis	[571]
		SPINK7, PPL, SEMA4B	Saliva	↓	Diagnosis	[572]
	miRNA	miR140‐5p, miR301a	Saliva	↓	Diagnosis	[572]
		miR‐181a‐1	Plasma	↓	Diagnosis	[570]
		miR‐627, miR‐629, miR‐652	Plasma	↑	Diagnosis	[573]
	lncRNA	lncUEGC1	Plasma	↑	Diagnosis	[574]
ESCC	miRNA	miR‐21	Plasma	↑	Prognosis prediction	[575]
	miRNA	miR‐106a, miR‐18a, miR‐20b, miR‐486‐5p, miR‐584	Plasma	↑	Diagnosis	[576]
		miR‐223‐3p	Plasma	↓	Diagnosis	[576]
		miR‐20b‐5p, miR‐28‐3p, miR‐192‐5p, miR‐223‐3p, miR‐296‐5p	Serum	↑	Diagnosis	[577]
	lncRNA	HOTAIR	Serum	↑	Diagnosis	[578]
		POU3F3	Plasma	↑	Diagnosis	[579]
HCC	mRNA	TGF‐β1, GP73	Serum	↑	Diagnosis	[580]
		ER‐α36	Serum	↓	Diagnosis	[581]
	lncRNA	lncRNA–UCA1, lncRNA–WRAP53	Serum	↑	Prognosis prediction	[582]
		HULC, Linc00152	Plasma	↑	Diagnosis	[583]
OSCC	mRNA	IL8, IL1B, DUSP1, HA3, OAZ1, S100P, SAT	Saliva	↑	Diagnosis	[584]
	miRNA	miR‐200a, miR‐134	Saliva	↑	Diagnosis	[585]
		miR‐1290	Plasma	↓	Diagnosis	[586]
	lncRNA	HOTAIR	Plasma	↑	Diagnosis	[587]
PCa	miRNA	miRNA‐375, miRNA‐141	Serum	↑	Diagnosis and prognosis prediction	[588]
	lncRNA	lncAPP	Urine	↑	Prognosis prediction	[589]
		lincRNA‐p21	Urine exosomes	↑	Diagnosis and prognosis prediction	[590]
		lncRNA FR0348383	Urine	↑	Diagnosis	[591]
		PCAT18	Plasma	↑	Prognosis prediction	[592]
		SAP30L‐AS1, SChLAP1	Plasma exosomes	↓	Prognosis prediction	[593]
TNBC	miRNA	miRNA‐155, miRNA‐21	Serum	↑	Diagnosis	[594]
		miRNA‐205	Serum	↓	Diagnosis	[594]

Abbreviations: BC, breast cancer; CRC, colorectal cancer; ESCC, esophageal squamous cell carcinoma; GC, gastric cancer; HCC, hepatocellular carcinoma; OSCC, oral squamous cell carcinoma; PCa, prostate cancer; TNBC, triple‐negative breast cancer.

### 6.2 RNA Regulatory Networks and Prognostic Prediction

With the rapid development of high‐throughput sequencing technology and bioinformatics in recent years, RNA regulatory networks indicators have become more important in studying disease prognosis assessment. Regulatory networks of RNA molecules not only participate in disease development through mechanisms (e.g., posttranscriptional regulation and epigenetic changes), but also their expression profile characteristics may provide a crucial tool for guiding clinical decision‐making.

The poor prognosis of tumor cells that are related to immune escape function and drug resistance are regulated by RNA networks through various means. Regarding immune escape: in NSCLC, circ_0074158 upregulates the expression of the RBP QKI6 by direct binding, thereby promoting the STUB1 (an E3 ubiquitin ligase)‐mediated ubiquitinative degradation of PD‐L1. At the same time, it induces CD8+ T cells to secrete IFN‐γ and TNF‐α, interfering with immune escape capacity [595]; circ_0000512 has the exact opposite effect in TNBC—it sequesters miR‐622 to upregulate the expression of its target gene CMTM6, inhibiting PD‐L1 ubiquitinative degradation and strengthening the tumor immunosuppressive microenvironment [596]. The m6A reader protein YTHDF2 is also involved in immune escape regulation: YTHDF2 upregulated in PC regulates CD8+ T cell infiltration‐related gene network, affects immune checkpoint molecules expression, promotes tumor immune escape, which relates to poor prognosis [597]. Regarding drug resistance: the lncRNA SNHG12 is overexpressed in renal cell carcinoma. It binds to the transcription factor SP1 and inhibits its ubiquitinative degradation, thereby promoting the transcriptional activation of its target gene CDCA3. Ultimately, this enhances tumor cell resistance to sunitinib, and patients with high SNHG12 expression have poorer clinical prognosis [598]; in NSCLC, lncRNAs can regulate EGFR‐TKIs resistance‐related pathways through the ceRNA mechanism. Some lncRNAs can also form complexes with RBPs, synergizing with m6A modification to alter the stability of target mRNAs and accelerate the enrichment of drug‐resistant subpopulations [599]. The crosstalk between these mechanisms directly affects clinical treatment response. For example, m6A subtype classification shows that BLCA patients with high expression of m6A regulators such as METTL3 have enriched inhibitory immune components in the TME, lower response rates to chemotherapy and immunotherapy, and significantly shortened overall survival [600].

ncRNAs demonstrate unique advantages in the prognostic evaluation of cancer. Research has shown that miR‐132 acts as a tumor suppressor, with its downregulation due to hypermethylation serving as a marker of poor prognosis in CRC patients [32]. Furthermore, the lncRNA DANCR is upregulated in CRC tissues and is associated with TNM stage, histological grade, lymph node metastasis, shorter overall survival, and disease‐free survival [601]. Additional studies have identified specific miRNAs as novel biomarkers for resistance to particular chemotherapy regimens, with alterations in their expression levels correlating with patient survival rates. For instance, miR‐4299 and miR‐196b have been identified as potential biomarkers for chemotherapy resistance in patients with intestinal adenocarcinoma [602, 603]. Moreover, advancements in liquid biopsy technology have broadened the application of RNA biomarkers in clinical prognostic evaluations [538].

RNA modifications and their regulators are frequently aberrantly expressed in tumor tissues and are closely associated with the prognosis of cancer patients [349]. Studies have shown that m5C RNA methyltransferases hold significant potential for prognostic prediction in cancer [604]. In clear cell renal cell carcinoma, the mRNA levels of NOP2 and NSUN4 in tumor tissues are elevated compared with those in normal tissues, while the mRNA levels of NSUN6 and the m5C eraser TET2 are reduced. These four m5C regulators constitute a risk signature for determining patient prognosis [605]. In PC, the levels of NSUN6 are significantly reduced, and elevated NSUN6 expression is associated with a lower risk and improved prognosis for PC patients [606]. Additionally, the m1A modification status in RNA molecules can also be used to predict patient prognosis and even serve as prospective therapeutic targets [349]. Studies have identified a negative correlation between ALKBH1 overexpression and overall survival in CRC patients. Conversely, decreased ALKBH3 expression in CRC is associated with poorer overall survival [607]. Small‐molecule inhibitors under development that target RNA modification sites and RNA‐modifying enzymes can offer new, more targeted ways to combat cancer. Moreover, sometimes, a single enzyme can have an opposite effect in different cancers, or even have different effects in the same cancer. As a result, more studies are required to confirm and to clarify the mechanisms of RNA methylation in cancer and to provide a better explanation for the existing conflicting studies, and also address the discrepancies in the literature.

The most important part of any cancer diagnosis, molecular subtype, and prognosis is the RNA expression profile and modification status. The unique invaluable value of these tools in early screening, monitoring of therapeutic efficacy, and prognostic prediction, especially the advantage of noninvasive liquid biopsy. It provides a new pathway for precision medicine in cancer. There are many issues that need urgent resolution. For example, first, the biomarkers have imbalanced sensitivity and specificity. Further, there is no standardization of detection. The tissue tracing is also difficult. Also, there is no multicenter large‐sample validation. Further research efforts would shift from the single‐biomarker paradigm to a multidimensional RNA panel construction combined dynamic monitoring systems with nanotechnology for maximum detection efficiency. This will facilitate the transfer of RNA‐based diagnostics from the laboratory to the clinic for accurate cancer diagnosis and personalized prognostic management.

## 7 Cancer Therapeutic Strategies Targeting RNA Regulatory Networks

Traditional cancer therapies like surgery, radiotherapy, and chemotherapy can delay cancer recurrence and extend patient survival for a time. However, frequent tumor recurrence and drug resistance are often the main reasons for poor prognoses. Also, as these chemotherapies and radiotherapies are nonspecific, they also cause major toxic side effects and sometimes even death. In this context, precision medicine has emerged as a pivotal direction in medical research. The therapeutic potential of RNA has been extensively studied, with mRNA vaccines, shRNAs, small interfering RNAs (siRNAs), antisense ASOs, RNA aptamers, and CRISPR technology becoming prominent research hotspots.

### 7.1 | mRNA Vaccine

mRNA vaccines have emerged as a promising platform for cancer immunotherapy [608]. mRNA‐based cancer vaccines function by encoding tumor‐specific antigens, which are then translated into proteins by the patient's cells—particularly antigen‐presenting cells—thereby stimulating innate/adaptive immune responses against cancer [609]. Owing to advantages such as high efficacy, safe administration, great development potential, and low production costs, mRNA cancer vaccines outperform other traditional vaccine platforms [610]. Recently, the United States Food and Drug Administration (US FDA) approved lipid nanoparticles (LNP)‐loaded mRNA vaccines for COVID‐19 prevention, and mRNA cancer vaccines have achieved favorable therapeutic outcomes in multiple clinical trials targeting various aggressive solid tumors [608].

However, relatively few mRNA vaccines for tumors have advanced to Phase III clinical trials, which may be attributed to the following reasons. First, mRNAs encoding a single tumor antigen may induce tumor immune escape mutations; thus, vaccines targeting multiple tumor‐associated antigens or tumor‐specific antigens simultaneously need to be developed. Second, the heterogeneity of tumor surface antigens calls for a personalized vaccine design; however, their high production costs hinder its widespread application. The third factor emphasizes the effectiveness of adjuvants in immune responses. In other words, the TME can impair neoantigen recognition and immune cell function. The effects of the inborn instability, low delivery efficiency, and the poor transfection efficacy of mRNA itself restrict the therapeutic effect. Therefore, a continuous optimization of mRNA molecular structure and delivery vectors is in demand. Fifth, tumor patients often have diminished immune responses because they are old, taking other drugs, or have comorbidities, and these may require careful observation of other adverse reactions while boosting immunity [611]. Ultimately, however, advanced tumors are unlikely to be cured by mRNA vaccines alone; these should be combined with other treatments, like adoptive cell therapy and immune checkpoint blockade, to provide synergistic effects [611].

Strategies to improve mRNA vaccines focus on three key elements: mRNA sequences, delivery vectors, and adjuvants [611]. The use of nucleoside modification technology results in lower levels of immunogenicity in mRNA and a more efficient expression of proteins. Furthermore, the optimization of the polyadenylate tail structure improves stability and translational efficiency [612]. Innovations in adjuvants have focused on TLR agonists (e.g., Pam2Cys) incorporated into delivery systems or direct construction of mRNA or mRNA fused to the C3d adjuvant [613, 614]. Concerning delivery systems, while LNPs can shield mRNA from degradation, their cationic nature may harm cells. Therefore, making biodegradable lipids is an important direction. Furthermore, the use of stimulus‐responsive nanoplatforms enables targeted delivery in response to microenvironment‐specific stimuli—for instance, pH or enzyme activity or surface modification—and greatly enhances tissue specificity while mitigating off‐target toxicity [615].

### 7.2 Antisense Nucleic Acid Technology

Antisense nucleic acid technologies, such as ASOs and siRNAs, have demonstrated substantial potential in targeting oncogenic RNA molecules. ASOs are artificially produced single‐stranded DNA or RNA molecules of 15–25 nucleotides in length and bind to the target mRNAs or ncRNAs due to base complementarity. This binding either inhibits their translation or leads to their degradation, thus controlling gene expression and RNA behavior. The role of this tech is an “antisense” sequence that binds to target RNA. This is useful for either silencing the genes responsible for cancer and regulating splicing or correcting the function of RNA [34]. Targeting aberrantly expressed RNAs using ASOs may represent a promising strategy for cancer treatment. Numerous studies have confirmed that ASO‐mediated knockdown of metastasis‐associated LUAD transcript 1 (MALAT1) significantly inhibits tumor growth and metastasis in BC and LC [616, 617]. Additionally, ASO‐mediated downregulation of lncRNA–SChLAP1 effectively restricts tumor formation and metastasis in PCa patients exhibiting high expression of this molecule [618]. Furthermore, locked nucleic acid‐modified antisense oligonucleotides (LNA‐ASOs) targeting lnc‐USMycN significantly suppress tumorigenesis in glioma mice models [619]. Lastly, treatment with Stat3‐ASO reduces circulating VEGF, basic FGF, tumor volume, and weight in HCC [620].

Compared with siRNAs, a significant advantage of ASOs is their ability to target immature miRNAs in the nucleus, facilitated by the ubiquitous distribution of RNase H, which is recruited by ASOs in both the cytoplasm and nucleus. In contrast, siRNAs, which depend on the RISC that is present only in the cytoplasm, can only act on mature miRNAs [621]. However, the inherent limitations of ASOs have hindered their clinical translation. ASOs have high negative charges and are easily degraded by enzymes and eliminated from circulation too quickly—these factors hamper their use in the clinic [622]. The efficient delivery of ASO drugs to the right tissues and subcellular targets is hampered by several physiological barriers. ASOs used for tumor therapy are given intravenously, and they must overcome the vascular endothelial barrier to reach the TME. While chemical changes can improve the pharmacokinetics, biodistribution, and targeting of new ASOs, effective delivery systems are still required to further enhance their therapeutic effect. At present, nucleic acid delivery systems like LNPs, liposomes, polymer nanoparticles, and bioconjugates have been employed in research, with an increasing number of ASO‐based therapies undergoing clinical trials. There is a growing need for drug delivery systems capable of carrying, protecting and targeting ASOs in tissues for the efficient clinical implementation of ASOs [623].

The role of siRNAs is not just limited to studying single‐gene functions in vitro and in vivo but they also have therapeutic potential with distinct advantages. They can target “undruggable” entities in cancer and other diseases [624]. siRNA consists of chemically synthesized double‐stranded RNA (dsRNA) that usually comprising of 19 to 23 nucleotides in length with the presence of 2‐nucleotide overhang at the 3’ end, also having a structure similar to that of endogenous miRNAs. The mechanism of action of siRNAs begins with recognition and processing by the Dicer enzyme. Then they are unwound by AGO proteins. Here, the guide strand is incorporated into the RISC and the passenger strand gets degraded by exonucleases. Finally, the binding of the RISC‐siRNA complex facilitates the cleavage and degradation of target RNAs. This occurs by binding of the guide strand to complementarily binding target RNAs. siRNAs and miRNAs are mechanistically similar but also differ in several important regards: siRNAs bind to their targets with near perfect sequence complementarity and induce cleavage of the target RNA, while miRNAs function by repressing translation. siRNAs have become indispensable tools for studying single‐gene functions because they can precisely silence target gene expression in a sequence‐specific manner [625]. The actions of siRNAs have persistence and a cascading amplification effect. RISC can continuously target mRNAs, leading to a prolonged duration of drug action on the one hand [106, 626]. On the other hand, the sense strand released by siRNAs (or its degradation products) can function as a primer for amplification of new dsRNAs using target mRNAs and RNA‐dependent RNA polymerases as catalysts. After this, Dicer degrades these dsRNAs into siRNAs again to get back into the RNA interference cycle and achieve cascade amplification of the drug effect [627, 628]. The continuous presence of siRNA molecules amplifies their potency or biological value. Such processes, known as cascade amplification processes, will strengthen the value of siRNA. This not only improves siRNA efficacy in gene silencing but also offers a basis to translate siRNA into clinical therapeutic applications. Moreover, such amplifying processes could allow siRNA to find application in pathologies with low dosages. The authors also demonstrated that specific siRNAs encapsulated in polyethyleneimine (PEI) target circITGB6 to inhibit liver metastasis [488]. In cancers such as NSCLC, therapeutic siRNAs designed to target molecular markers associated with tumor development can significantly inhibit tumor growth [629].

The use of siRNAs in the clinic faces two main challenges: immunogenicity and off‐target effects [630]. In terms of the immune response, Toll‐like receptors are able to recognize siRNAs. The structural similarity of the siRNAs with that of the viruses’ dsRNAs allows for the triggering of excessive innate immune responses and the uncontrolled release of cytokines. To tackle the issue, chemical modifications, for example, 2′O‐methyl modification can, are effective in TLR activation inhibition. Moreover, designing the sequences with known immune‐stimulatory motifs such as the UG dinucleotide and 5′‐UGU‐3′ motifs can avoid these [631, 632]. About off‐target effects, siRNAs may cause off‐target gene silencing by fully saturating endogenous RNAi machinery or by partial complementarity to 3′UTRs, thus showing miRNA‐like gene silencing [633]. New control designs with guide strand point mutations such as 2‐nucleotide mutations can increase the specificity of experiments [630]. Although siRNA therapies face many obstacles in the treatment of cancer, their in vivo delivery technology is rapidly advancing, thereby representing an exciting and novel area of biomedicine and drug development [634].

### 7.3 RNA Aptamers and Nanotechnology

**FIGURE 5:**
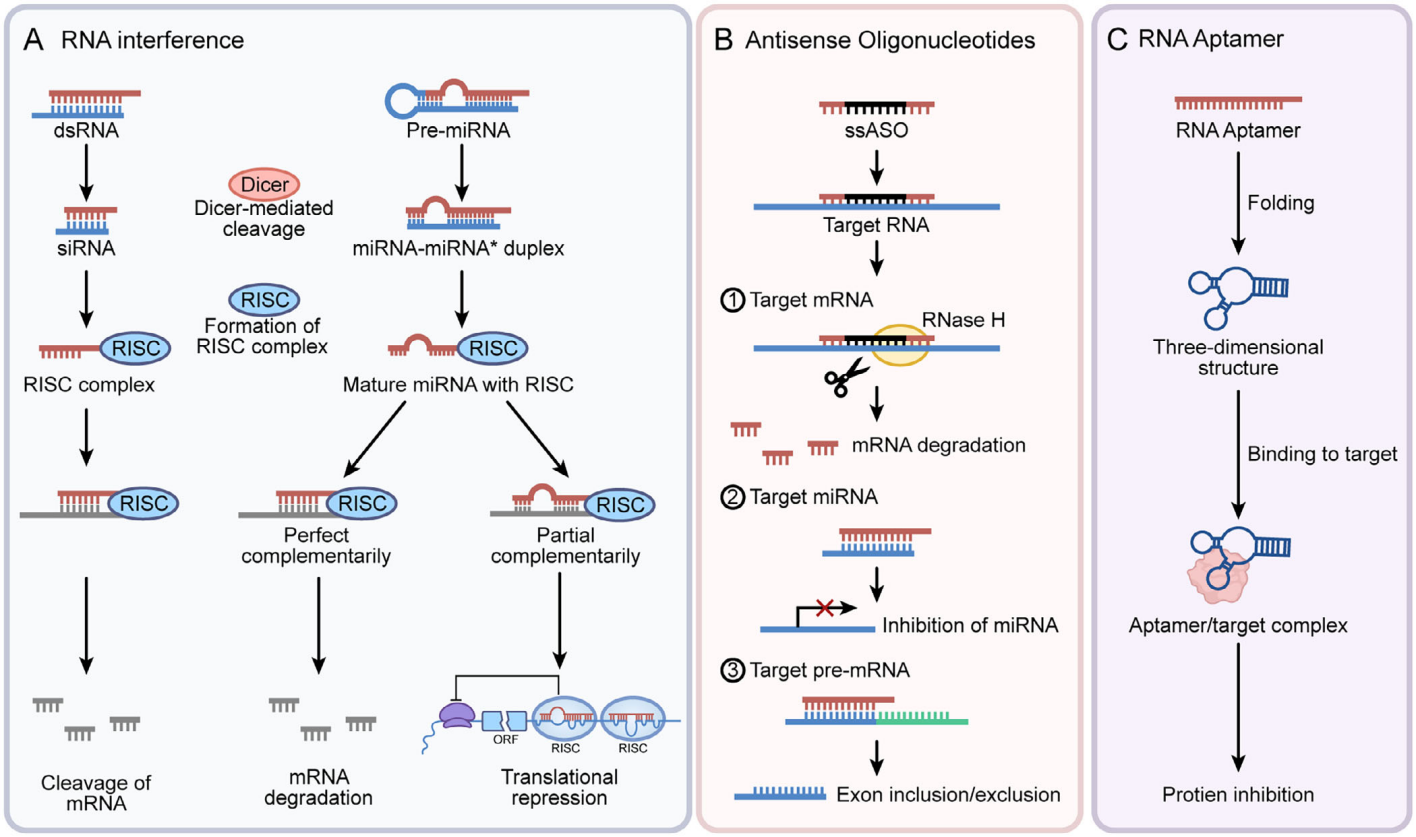
Mechanisms of action of common RNA therapies. (A) siRNA is recognized by the Dicer enzyme and subsequently processed. The double‐stranded siRNA is unwound through the helicase activity of antisense oligonucleotide (AGO). One guide strand remains in the RISC, while the passenger strand is degraded by exonucleases. The RISC–siRNA complex then mediates the cleavage and degradation of the target RNA. This process is analogous to the action mechanism of miRNAs; unlike miRNAs, siRNAs bind to sequences with perfect or nearly perfect complementarity, leading to target cleavage rather than translation inhibition. (B) ASOs bind to target mRNAs or ncRNAs through base complementary pairing, effectively blocking their translation or promoting their degradation, thus regulating gene expression or RNA function. (C) RNA aptamers adopt various secondary and tertiary structures and form unique three‐dimensional conformations upon binding to target molecules, which confers high specificity and affinity. Following binding, RNA aptamers can obstruct interaction between target molecules and other entities through steric hindrance or alter the conformation of target molecules, resulting in the loss of biological activity.

RNA aptamers are functional RNAs that can specifically bind to target molecules. Right now, almost all RNA aptamers come from in vitro selection methods, particularly the systematic evolution of ligands by exponential enrichment (SELEX) [635]. These molecules can form complex secondary and tertiary structures that include stems, loops, hairpins, triplexes, and quadruplexes. The processes by which they interact with their targets that include everything from a small‐molecule compound, metal ions to proteins and even cell through hydrophobic interaction, hydrogen bonding, and electrostatic interaction to form three‐dimensional complexes having high specificity and affinity. RNA aptamers are more specific and have a greater affinity for RNA secondary structures than complementary oligomers [636]. Furthermore, aptamers can not only degrade RNA but also inhibit RNA functions, highlighting their potential as therapeutic agents targeting RNA. The working mechanisms of the three RNA‐based therapeutic approaches are shown in Figure 5.

RNA aptamers are capable of binding to wide‐ranging targets having a capacity that holds greater promise than others for cancer diagnosis and prognosis, as well as therapy [35]. They are sensitive detectors of serum levels of VEGF, which is a major regulator of angiogenesis, widely employed as a biomarker for BC, LC, and CRC [637]. In the diagnosis and treatment of CRC, an increasing number of RNA aptamers have been confirmed to have considerable clinical value. Using tumor‐bearing mouse models, many nuclease‐resistant RNA oligonucleotides have been screened, and the study was able to identify RNA aptamers that selectively localize to hepatic metastatic lesions of colon cancer. One of the aptamers specifically binds to the RNA helicase p68 that is overexpressed on CRC [638]. RNA aptamers can target both the cell surface marker and important intracellular component (such as proteins). For instance, an aptamer that binds to the RNA helicase DHX9 can selectively target cancer cell nuclei in vivo, thus providing a feasible strategy for nucleus‐targeted delivery [639]. Besides their potential to target the desired site, RNA aptamers can also act as antagonists. They can prevent the interaction between an extracellular target and its ligand [35]. The carcinoembryonic antigen (CEA) overexpression in CRC cells increases the cell adhesion and the resistance to anoikis and the liver metastasis [640]. An RNA aptamer selective to CEA, screened during SELEX, will bind the metastasis‐related domain of CEA. As a result, it blocks the interaction between CEA and the heterogeneous nuclear ribonucleoprotein M4 or death receptor 5. This has significant impact on colonic cancer cells’ liver metastasis in mice [641].

**FIGURE 6:**
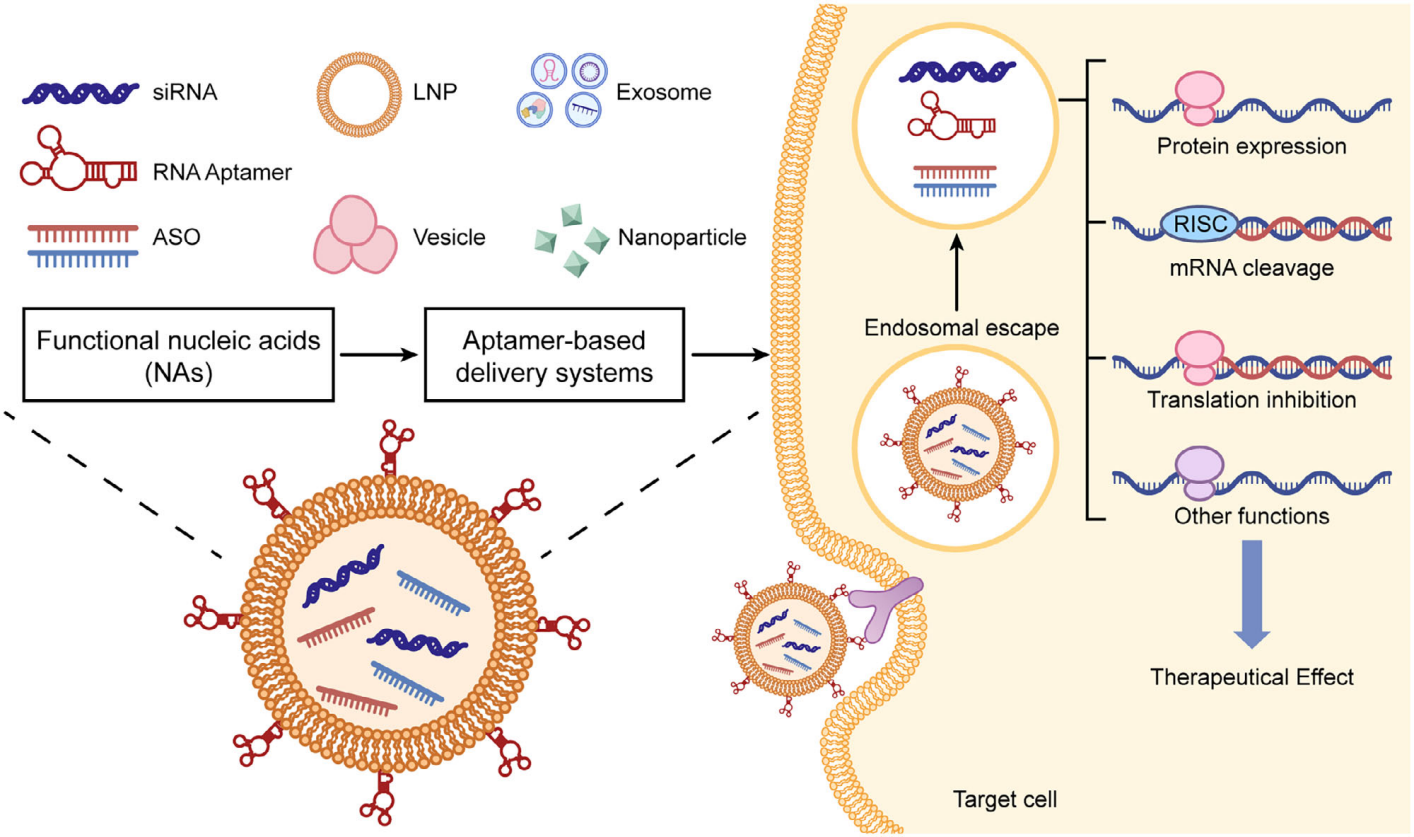
Targeted delivery of functional nucleic acids based on nucleic acid aptamers. Nucleic acid aptamers, due to their high specificity and selective binding capabilities to cell surface proteins, function as molecular recognition units that precisely bind to target cells. This property facilitates the efficient transport of various functional nucleic acids, including siRNA and ASO. Furthermore, when combined with nanomaterials such as lipid nanoparticles and exosomes, aptamers can enhance loading capacity and delivery efficiency. This ensures that these functional nucleic acids accurately and efficiently reach specific cells or organs, thereby exerting effects such as regulating gene and protein expression, inhibiting tumor growth, and preventing abnormal cell proliferation and migration.

RNA aptamers are a new class of molecules with potential in diagnosis and therapy that work as antibodies but avoid many of their drawbacks, including large molecular weight, strong immunogenicity, and peptidase degradation [642, 643, 644]. RNA aptamers have significant advantages over protein‐based reagents due to the cell‐free assembly property, enabling their low‐cost, fast, and large‐scale manufacturing [645]. Aside from working alone, RNA aptamers are now increasingly being combined with nanotechnology to enhance their diagnostics [646, 647, 648]. Functionalized nanoparticles like liposomes can be aptamers, and they facilitate targeted drug delivery, improving bioavailability and reducing off‐target effects [649]. Nanostructures can be synthesized to encapsulate chemotherapeutics, siRNA or imaging probes, with aptamer modifications on their surface permitting recognition of surface markers specific to cancer (e.g., PSMA in PCa or HER2 in BC) [650, 651]. Furthermore, aptamer‐based biosensors employ nanomaterials like quantum dots and graphene oxide, which enable ultrasensitive detection in liquid biopsies of cancer biomarkers [652]. It is clear that RNA aptamers have been found to be a new molecular tool and are proving to be useful tools for more and more fundamental applications like bioanalysis and medical diagnosis as well as new areas like nano‐technology and gene therapy (Figure 6).

### 7.4 RNA Editing and Gene Therapy

CRISPR–Cas system is a revolutionary gene editing tool. The well‐studied CRISPR–Cas9 system targets DNA. This system can also be used for pathogen detection and antibacterial strategies besides for gene therapy [653, 654]. Recently, the RNA‐targeting CRISPR–C2c2/Cas13a system has attracted much scientific attention [655]. The CRISPR–Cas13a system recognizes and binds to single‐stranded RNA sequence in a specific manner, followed by cleavage of RNA in a nonspecific manner, thereby functioning as a molecular tool to disrupt gene expression or edit gene transcripts at the transcriptional level [656]. The RNA editing technology has unique potential in the cancer therapy field to target aberrant RNAs of oncogenes and repair critical mutations.

Solid tumor therapies value CRISPR–Cas13a as follows: Three aspects: first, it directly knocks down oncogenic transcripts. For instance, in case of NSCLC, targeting NSCLC‐associated circRNA C190 or gene EML4‐ALK can result in reduction of cell viability by 50% [36]. The delivery of CRISPR–Cas13a through an adeno‐associated virus (AAV) system can efficiently knock down the TERT/EZH2/RelA complex in HCC with a significant inhibition rate. One other important application is reversing therapeutic resistance [36]. This system can accurately identify and target mutant KRAS transcripts to overcome chemoresistance [657]. CRISPR–Cas13a is very helpful in therapeutic target with respect to oncogenes other than direct targeting. It is also able to help in immunomodulation and TME reprogramming. According to BLCA, the application of this technology seriously interferes with the proliferation, migration, and invasion of tumor cells and induces apoptosis. Moreover, this is achieved by knocking down eRNAs, (e.g., SMAD7e and P2RY2e), and lncRNAs, (e.g., GACAT3 and MALAT‐1) [36]. Thus, the multifaceted applicability of RNA editing suggests its utility not only in silencing driver oncogenes but also in reprogramming the immunosuppressive microenvironment. More research is still needed about the medicinal use of CRISPR–Cas13 in the treatment of nonsolid tumors [658].

Additionally, the Cas13a system can be combined with other devices to construct signal‐amplifying sensors for detecting RNA or exosomes. This system has also been proven to hold broad prospects in tumor diagnosis and early screening [659, 660]. For example, the CRISPR–Cas13a/Cas12a system was used to develop a fluorescent biosensor capable of simultaneously detecting BC biomarkers circROBO1 and BRCA1. The findings of the technology we developed recently of circROBO1 was instrumental in revealing the molecular mechanism by which circROBO1 via the ceRNA mechanism binds to miR‐498 to regulate expression of BRCA1. Our these observation will provides a crucial basis for an in‐depth understanding of the intrinsic regulatory network in BC [661]. The sensitivity, speed and minimally invasive nature of detection in nonsolid tumors leukemia and lymphoma, has advanced with CRISPR–Cas13 [658]. Traditional detection techniques are complex to operate and rely on sophisticated instruments; in contrast, CRISPR–Cas13 can accurately capture targets (e.g., leukemia fusion transcripts and lymphoma BRAF mutant RNAs) through RNA‐targeted recognition combined with a fluorescent reporting system [662]. At present, this technology has a very low limit of detection that can detect trace amounts of minimal residual disease that would not be picked up by standard techniques. This facilitates early diagnosis and assessment of treatment efficacy [663].

Unlike traditional genomic tools like CRISPR–Cas9, Cas13 regulates the gene expression by targeting intracellular RNA molecules without permanently altering the genomic DNA. This type of intervention, which is reversible and transient, greatly reduces the risk of off‐target‐induced irreversible mutations [664]. The Cas13 system is additionally not cytotoxic or procarcinogenic as it does not activate the p53 pathway, which gets activated upon DNA breaks. Therefore, it is a more versatile tool that can be used when further fine‐tuning of gene expression is required, rather than a full knockout of functions [665, 666]. More importantly, this technology can target molecular classes that are difficult to access with traditional DNA editing—such as noncoding RNAs and mitochondrial RNAs—greatly expanding the scope of transcriptome intervention [666]. Nonetheless, one should not lose sight of its limitations. The transient nature of RNA targeting means that its duration of action is limited by the half‐life of the target RNA and the cell's own transcriptional dynamics. This poses challenges of repeated administration in the treatment of diseases requiring long‐term phenotypic correction [667]. Though the use of RNA targeting does not present genomic integration‐associated risks, it also forfeits the possibility of achieving lifelong efficacy through a single intervention. This “reversibility” may instead result in burdening continuous intervention in chronic disease management [666, 667].

The challenges of the clinical translation of RNA editing therapies are standing with the promising preclinical experiments and high rates of inhibition. A major obstacle is the development of safe and effective in vivo delivery systems that can achieve targeted tissue distribution while minimizing off‐target accumulation [668]. The presence of immunogenicity and potential insertional mutagenesis is a risk in the use of viral vectors such as AAV. When it comes to nonviral delivery systems, these face issues of poor stability, low packaging efficiency, and potential cytotoxicity, especially in LNPs and polymeric nanoparticles [669]. The innate immunogenicity of Cas proteins from bacteria may activate the immune system of the host that could lessen their therapeutic effectiveness and elicit side‐effects [670]. Additionally, although the collateral cleavage activity of Cas13a can be used for signal amplification in diagnostics, it poses a risk of nonspecific RNA degradation in therapeutic contexts, which may disrupt normal cellular functions [671]. Currently, CRISPR–Cas13‐based tumor therapy is still in the preclinical and early clinical research stages; thus, data on its long‐term efficacy and safety remain limited and require further exploration.

Therefore, there is a need to design Cas13 variants with lower immunogenicity, optimize guide RNA design to minimize off‐target effects, and develop new delivery platforms [671]. Exosome‐based or targeted nanoparticle‐mediated delivery systems for CRISPR components can reduce immunogenicity while enhancing targeting ability, thereby improving therapeutic precision [672, 673]. To address the adverse cytotoxicity caused by the collateral RNA cleavage activity of CRISPR–Cas13, Xu et al. developed an ultra‐small RNA editing platform designed based on IscB (the evolutionary ancestor of Cas9). This platform exhibits RNA editing activity comparable to or higher than that of Cas13; it can effectively alter splicing outcomes in human cells and further mediate trans‐splicing to correct any mutations at the mRNA level, without inducing cytotoxicity [674]. Studies have confirmed that combining gene therapy with chemotherapy can effectively alleviate the limitations of single gene therapy in tumor suppression [675]. This indicates the great significance of developing chemotherapeutic drugs that integrate immune checkpoint inhibition, promote immune infiltration, and induce tumor cell death.

**FIGURE 7:**
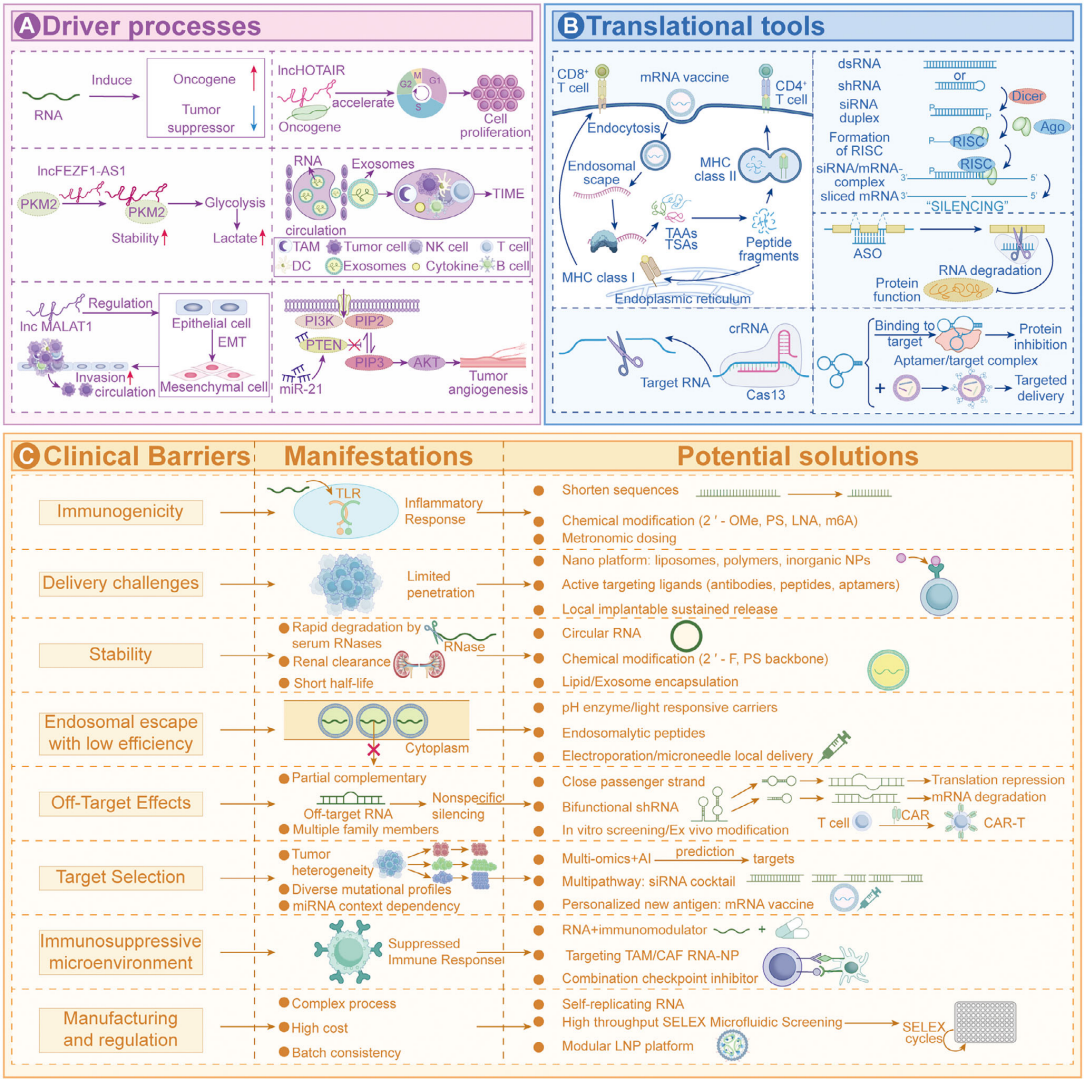
Integrated schematic of RNA regulatory networks—translational tools—clinical barriers. (A) Driving processes: RNAs regulate tumor progression through multiple processes, including inducing oncogene activation and tumor suppressor gene silencing, cell cycle dysregulation to promote tumor proliferation (e.g., lncRNA HOTAIR), tumor metabolic reprogramming (e.g., lncRNA FEZF1‐AS1), formation of the immunosuppressive microenvironment, invasion and metastasis (e.g., lncRNA MALAT1), and angiogenesis (e.g., miR‐21). (B) Translational tools: Based on the structural and functional characteristics of RNAs, various RNA‐based therapies have been developed, including mRNA vaccines, RNA interference therapies, antisense oligonucleotide therapies, RNA aptamers, and CRISPR–Cas13 technology. (C) Clinical barriers and potential solutions: Currently, the clinical application of RNA therapies still faces numerous challenges, such as immunogenicity, delivery challenges, stability, low‐efficiency endosomal escape, off‐target effects, target selection, the immunosuppressive microenvironment, and manufacturing and regulation. Multiple potential solutions have been explored to address the aforementioned challenges.

Currently, RNA cancer therapies exhibit a parallel development pattern of “personalization and combination” in clinical trials, demonstrating tremendous application potential (Table 5). Figure 7 illustrates RNA networks driving cancer processes, RNA‐based therapies, as well as the associated clinical translation barriers and potential solutions. mRNA vaccines are the only RNA cancer therapies that have entered Phase III clinical trials to date. Moderna's mRNA‐4157 has completed Phase IIb, confirming that combination with anti‐PD‐1 monoclonal antibody reduces the risk of recurrence/death in high‐risk melanoma by 49%. It has also been granted US FDA Breakthrough Therapy Designation, becoming the first personalized mRNA tumor vaccine to advance to Phase III (NCT05933577) [676]. Earlier technologies such as siRNA–LNPs and CRISPR–Cas13 remain in preclinical/Phase I stages. RNA aptamers are mostly used for targeted delivery [677]. Overall, mRNA vaccines have progressed from proof‐of‐concept to randomized controlled confirmation phases, while RNA interference and editing therapies still need to overcome bottlenecks in delivery and safety. In the future, researchers should develop solutions addressing the corresponding challenges of RNA therapies, and advance RNA cancer treatment into clinical practice through technologies centered on “target prediction‐precise delivery‐immune combination.”

**TABLE 5:** RNA‐based cancer therapies with clinical trial results (2024–2025).

Type of RNA therapy	Drug/technology name	Target/mechanism	Delivery system	Clinical trial phase	Efficacy assessment/risk monitoring	Clinical trial registration number	References
mRNA vaccine	mRNA‐4157 (V940) combined with Pembrolizumab	Personalized tumor neoantigens (up to 34 types)	mRNA‐4157: intramuscular injection; pembrolizumab: intravenous injection	Phase IIb	Compared with pembrolizumab monotherapy, mRNA‐4157 adjuvant therapy achieved longer RFS and a lower incidence of recurrence or death events. Most TRAEs were Grade 1–2. Grade ≥3 TRAEs occurred in 25% of patients in the combination group and 18% in the monotherapy group; no mRNA‐4157‐related Grade 4–5 events were reported. The frequency of IMAEs was similar between the two groups.	NCT03897881	[678]
mRNA vaccine	WT1‐mRNA/DC	WT1 (glioblastoma multiforme, malignant pleural mesothelioma, metastatic breast cancer, and other solid tumors)	Intravenous infusion of autologous DCs ex vivo loaded with WT1‐mRNA	Phase I/II	Could induce WT1‐specific CD8^+^ T cell responses; patients with type 1 T cell responses (IFN‐γ^+^) had significantly prolonged mOS (HR 0.48, *p* < 0.01); only Grade 1–2 local/systemic adverse reactions were observed.	NCT01291420	[679]
mRNA	Autogene cevumeran	Predominantly somatic passenger mutations	Intravenous injection of liposomes	Phase I	At a median follow‐up of 3.3 years, vaccine‐expanded CD8^+^ T cells remained renewable; 50% of patients developed mutation‐specific long‐lived memory T cells; mPFS was 16.3 months, and mOS was 34.5 months; only Grade 1 flu‐like symptoms and injection site reactions were reported.	NCT05968326	[680]
Antisense oligonucleotides	AZD8701	Effectively reduces FOXP3 expression and reverses the immunosuppressive function of human Tregs in primary cells	Subcutaneous injection	Preclinical study (Phase Ia/b clinical trial ongoing)	FOXP3 ASO reduced FOXP3 levels in Tregs by over 70% in vitro and in vivo, upregulated Treg effector molecules (e.g., ICOS, CTLA‐4, CD25, and 4‐1BB), enhanced CD8^+^ T cell activation, and exerted antitumor activity in syngeneic tumor models. Combination of FOXP3 ASO with immune checkpoint blockade further enhanced antitumor efficacy.	NCT04504669	[681]
siRNA	iExoKrasG12D	KRAS (pancreatic cancer)	Exosome encapsulation; intravenous administration	Phase Ib	The primary outcome of iExoKrasG12D showed no DLT, and the MTD was not reached even at the highest dose. In some cases, iExoKrasG12D therapy was associated with stable disease response (secondary outcome). Combination therapy of iExoKrasG12D with anti‐CTLA‐4 antibody (but not anti‐PD‐1) exhibited potent preclinical antitumor efficacy by enhancing CD8^+^ T cell antitumor activity.	NCT03608631	[682]
dsRNA	BO‐112	Mimics double‐stranded RNA and activates innate immunity	PEI‐NPs; intratumoral injection	Phase II	For PD‐1‐resistant advanced melanoma, combined with Pembrolizumab, the ORR was 25%, mPFS was 3.7 months, and safety was manageable.	NCT04570332	[683]
circRNA	circCDUPRT/circILNb	Encodes CDUPRT suicide gene and IL‐15 cytokine, respectively	LNP encapsulation; intratumoral injection	Preclinical study	For advanced melanoma, achieved sustained intratumoral expression in animal models; combined with prodrug 5‐FC showed significant antitumor efficacy and activated CD8^+^ T cells and NK cells.	—	[684]

Abbreviations: 5‐FC, 5‐fluorocytosine; AZD8701, FOXP3‐targeting antisense oligonucleotide; CDUPRT, cytosine deaminase‐uracil phosphoribosyltransferase; CTLA‐4, cytotoxic T‐lymphocyte‐associated protein 4; DC, dendritic cells; DLT, dose‐limiting toxicity; dsRNA, double‐stranded RNA; IL‐15, interleukin‐15; IMAE, immune‐mediated adverse event; LNP, lipid nanoparticle; mOS, median overall survival; mPFS, median progression‐free survival; MTD, maximum tolerated dose; NK, natural killer; ORR, objective response rate; PD‐1, programmed cell death protein 1; PEI‐NP, polyethyleneimine nanoparticle; RFS, recurrence‐free survival; TRAE, treatment‐related adverse event; WT1, Wilms tumor 1.

## 8 Discussion

RNA regulatory networks serve as the central hub for decoding cancer biology. Their dynamic complexity has not only reshaped our understanding of tumorigenesis and progression but has also propelled a shift in cancer diagnosis and treatment toward a focus on “network targeting.” Present researches have been incrementally revealing that the ncRNAs, which include miRNAs, lncRNAs and circRNAs, create a complex regulatory network that activates oncogenes by silencing tumor suppressor genes through multilevel mechanisms. These mechanisms consist of ceRNA sponge effect, RNP complex assembly and modification. Most RNAs do not function in isolation in biological processes. Instead, RNAs represent critical branch points in regulatory networks. The regulatory functions of pluripotent transcription factors are highly pleiotropic by virtue of their large networks of signal outputs that they dynamically impose on cellular transcriptomes and proteomes, which, in turn, determine cellular biological fates. A wide variety of RNA molecules that have pleiotropic regulatory functions interact. The emergence of regulatory interplay across regulatory paradigms weaves an exceptionally complex web of gene regulatory networks and establishing RNA regulatory networks as the wire frame of tumor cell heterogeneity, adaptive evolution, and therapeutic resistance.

Currently, the multidimensional roles of RNA regulatory networks in cancer initiation, progression, diagnosis, and therapeutic intervention have become increasingly prominent, demonstrating unprecedented value in clinical translation. However, existing research continues to face three major cognitive gaps: First, most mechanistic studies focus on individual RNA types or static interaction patterns, lacking a spatiotemporal dissection of dynamic transcriptome–epigenome–proteome networks. For instance, while RNA crosstalk between immune cells and cancer cells in the TME has been established, the coordinated regulation of this process by RNA modifications (e.g., A‐to‐I editing) requires further investigation. Second, the clinical translation of circulating RNAs in liquid biopsy encounter a “specificity bottleneck.” The sensitivity and tissue traceability of existing biomarkers in early screening need improvement; for example, some miRNA‐based liquid biopsy panels exhibit variable false‐positive rates across different patient cohorts, and currently US FDA‐approved detection methods have limitations in detecting early‐stage cancers or distinguishing malignant from benign nodules. Multidimensional diagnostic models should be developed by integrating single‐cell sequencing and spatial transcriptomics. Third, RNA‐targeted therapeutic strategies (e.g., ASOs, CRISPR–Cas13) show promise in precise intervention; however, the immunogenicity, tissue targeting efficiency, and off‐target effects of delivery systems remain key barriers to clinical application. For example, systemically administered ASOs and siRNA‐loaded nanoparticles typically exhibit suboptimal tumor biodistribution and may trigger innate immune responses via pattern recognition receptors. Likewise, the in vivo delivery of CRISPR–Cas13 components is limited by packaging capacity and vector immunogenicity, as well as collateral RNA cleavage. New solutions are being sought to guide drug delivery. They include biomimetic nanoparticles, engineered exosomes and cell‐specific ligand conjugation. This will need innovation across materials science, bioengineering, and biology.

Future research on RNA regulatory networks in cancer is expected to open many more exciting directions. First, we must advance past simplified studies of RNA species and move toward more integrated systems biology studies of the RNA species. The ultimate goal is to develop computational models that integrate multiomics transcriptomics, epitranscriptomics, and proteomics data to build dynamic maps of RNA interaction networks across cancer stages and subtypes. Second, solving the delivery barriers for RNA‐targeted therapeutics (ASOs, siRNAs, CRISPR–RNA editors) is vital. Further studies should focus upon delivery platforms of the next generation like tunable LNPs, programmable exosomes as well as ligand–receptor targeted conjugates, and their pharmacokinetics, biodistribution, and immunogenicity through rigorous preclinical studies. Third, the next step is to find and validate more robust biomarkers that will be able to improve the clinical utility of liquid biopsy RNAs. A recommended strategy is to combine different RNA types (e.g., miRNAs, circRNAs and exosomal RNAs) with other analytes (e.g., circulating tumor DNA, proteins) in a single panel that improves sensitivity and specificity and helps in tissue‐of‐origin determination. Finally, long‐term studies are essential to understand the potential mechanisms of acquired resistance to RNA‐targeted therapies and to develop effective combination strategies with immunotherapies, targeted therapies, or conventional treatments for durable clinical responses.

As research progresses, RNA regulatory networks will bridge the gap between gene regulation and phenotype expression, transforming cancer diagnosis and treatment from “targeting target” to “systemic remodeling.” Such an evolution will open new paths to address issues like tumor heterogeneity and metabolic reprogramming, and allow for a shift from intervening in disease to regulating life itself.

## Author Contributions

Xuan Yin, Zengkan Du, Shuya Jiang, Yan Liao, Changli Wang, Jiaqi Li, and Wangzheqi Zhang contributed to the manuscript writing and figure preparation. Xuan Yin, Zengkan Du, and Wangzheqi Zhang designed the work. Zui Zou, Haoling Zhang, Ting‐Ting Wei, and Wangzheqi Zhang supervised the work. All authors have read and approved the article. All authors read and approved the final manuscript.

## Funding

This study was funded by the Natural Science Foundation of China (82572494 and 82202584) and Shanghai Oriental Talent Top Project and Changhai Hospital Anesthesia Specialty Platform Construction Project.

## Ethics Statement

The authors have nothing to report.

## Conflicts of Interest

The authors declare no conflicts of interest.

## Data Availability

Data sharing not applicable to this article as no datasets were generated or analyzed during the current study. All information is derived from publicly available articles and datasets.
